# Tumor microenvironment responsive Mn_3_O_4_ nanoplatform for in vivo real-time monitoring of drug resistance and photothermal/chemodynamic synergistic therapy of gastric cancer

**DOI:** 10.1186/s12951-022-01441-6

**Published:** 2022-05-23

**Authors:** Hanrui Li, Xiaoxia Cai, Tong Yi, Yun Zeng, Jingwen Ma, Lei Li, Liaojun Pang, Na Li, Hao Hu, Yonghua Zhan

**Affiliations:** 1 Engineering Research Center of Molecular & Neuro Imaging of the Ministry of Education, School of Life Science and Technology, Xidian University, Xi’an, 710126 China; 2 Radiology Department, Ninth Affiliated Hospital of Medical College of Xi’an Jiaotong University, Xi’an, 710054 China; 3 Endoscopic Center of Zhongshan Hospital, Fudan University, Shanghai, 200032 China

**Keywords:** Gastric cancer, Multidrug resistance, Fenton-like reaction, Chemodynamic therapy, Photothermal therapy, Self-enhanced nanoplatform

## Abstract

**Background:**

Postoperative chemotherapy for gastric cancer often causes multidrug resistance (MDR), which has serious consequences for therapeutic effects. Individualized treatment based on accurate monitoring of MDR will greatly improve patient survival.

**Results:**

In this article, a self-enhanced Mn_3_O_4_ nanoplatform (MPG NPs) was established, which can react with glutathione to produce Mn^2+^ to enhance T1-weighted magnetic resonance imaging (MRI) and mediate in vivo real-time MDR monitoring. In vitro MRI results showed that MRI signals could be enhanced in the presence of hydrogen peroxide and glutathione and at acidic pH. In vivo MRI results indicated that MPG NPs could specifically target MDR cells, thereby realizing real-time monitoring of MDR in gastric cancer. Furthermore, MPG NPs have good chemodynamic activity, which can convert the endogenous hydrogen peroxide of tumor cells into highly toxic hydroxyl radical through Fenton-like reaction at acidic pH to play the role of chemodynamic therapy. In addition, Mn_3_O_4_ can significantly enhance the chemodynamic therapy effect because of its good photothermal conversion effect. Furthermore, in situ photothermal/chemodynamic synergistic therapy obtained remarkable results, the tumors of the mice in the synergistic therapy group gradually became smaller or even disappeared.

**Conclusions:**

MPG NPs have good biocompatibility, providing a good nanoplatform for real-time monitoring and precise diagnosis and treatment of MDR in gastric cancer.

**Graphical Abstract:**

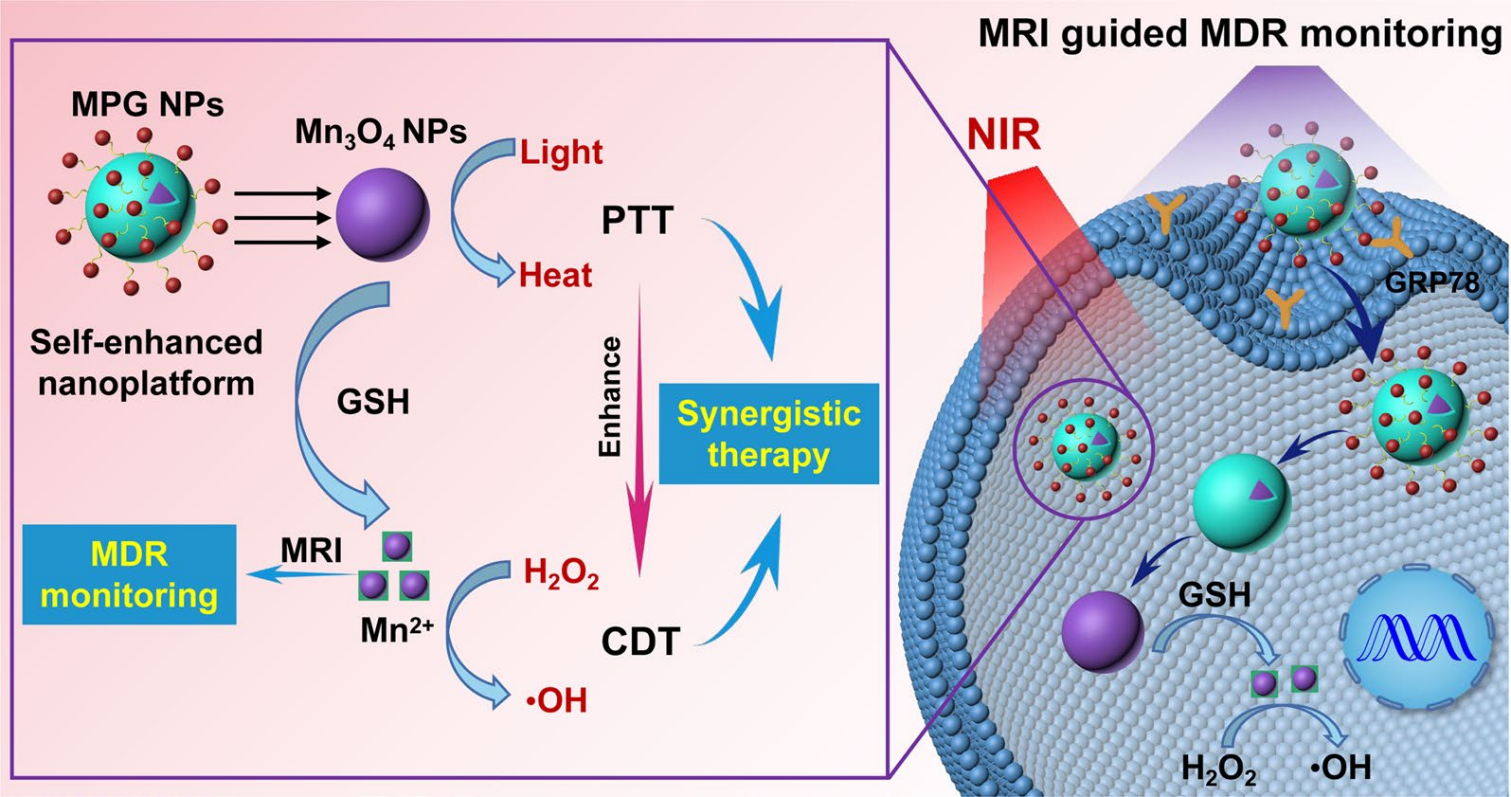

**Supplementary information:**

The online version contains supplementary material available at 10.1186/s12951-022-01441-6.

## Background

Gastric cancer is the fifth most common cancer in the world and seriously threatens human health [1]. Chemotherapy is the main adjuvant treatment after surgery. However, long-term chemotherapy easily induces multidrug resistance (MDR), which seriously influences treatment effects. Therefore, accurate monitoring of MDR is essential for the treatment of gastric cancer. In past studies, several MDR evaluation methods have been described, such as fresh tumor cell culture tests and cancer biomarker tests [2, 3]. These methods use fresh tumor cells to perform effective chemotherapy MDR detection in vitro or evaluate MDR through the expression of marker proteins. However, to a certain extent, such methods cannot reflect the real state of MDR. In addition, there have been studies using molecular imaging technology to perform in vivo imaging and to evaluate the degree of MDR through changes in tumor size after drug treatment [4]. The noninvasiveness of molecular imaging offers a good way to monitor MDR. However, delayed anatomical changes often cannot evaluate MDR in real time. Therefore, it is urgent to explore a new method to monitor MDR. As a new type of specific peptide, GMBP1 can specifically bind to the GRP78 receptor overexpressed on the surface of MDR gastric cancer cells [5] and significantly downregulate the levels of eukaryotic translation initiation factor 4E (EIF4E) and C-terminal binding protein 2 (CTBP2), thereby regulating the MDR phenotype of gastric cancer [6], which has been preliminarily proven to have a good ability to detect MDR gastric cancer [7]. Once MDR is accurately detected, individualized treatment can be implemented for patients, which can greatly improve the postoperative survival rate of patients. The individualized treatment of MDR gastric cancer commonly used in the clinic includes replacing new chemotherapeutic drugs or using adjuvant radiotherapy. However, the high cost and long-term and uncertain therapeutic effects of new drug development, as well as the regional limitations and body damage of radiotherapy, result in many difficulties associated with the clinical treatment of MDR gastric cancer that cannot be ignored [8, 9]. Therefore, it is important to explore other effective treatments for MDR in gastric cancer.

Studies have shown that the mechanism of MDR is closely related to the tumor microenvironment (TME), and due to the complexity and diversity of MDR, tumor treatment strategies for the TME have been applied to combat MDR tumors [10]. Among them, inducing intracellular ROS production is an effective tumor treatment method. High levels of ROS cause oxidative damage to cells, leading to cell death [11]. The Fenton reaction can use a Fenton catalyst to oxidize metal ions and convert hydrogen peroxide (H_2_O_2_) with low endogenous reactivity into highly toxic hydroxyl radicals (·OH) under acidic conditions to kill tumor cells, which makes chemodynamic therapy (CDT) based on the Fenton reaction a new and thriving tumor treatment strategy with its noninvasiveness and high selectivity [12, 13]. At present, some nanoparticles based on metal ions (such as Fe^2+^, Mn^2+^, Cu^2+^, etc.) have been used as CDT agents to induce tumor apoptosis. Unfortunately, the effects of CDT based on these nanoparticles are often limited by poor catalytic efficiency and low H_2_O_2_ concentration in the TME. In addition, a large amount of GSH in tumor cells eliminates ·OH, which will greatly weaken the effect of CDT [12, 14]. Therefore, the development of new therapeutic agents that can increase the efficiency of CDT and reduce the content of GSH in cells is of great significance for improving the efficacy of CDT.

The rapid development of nanomaterials provides a new opportunity for precise diagnosis and individualized treatment of tumors, such as polymeric nanoparticles, liposome, metal nanoparticles, 3D printing nanomaterials [15–25]. Among them, manganese oxide nanoparticles (NPs) have become a good choice for tumor diagnosis and treatment due to their good biocompatibility, in vivo imaging performance and TME responsiveness [26]. For example, Lin et al. synthesized MnO_2_-coated mesoporous SiO_2_ for Fenton-like reaction Mn^2+^ delivery and GSH depletion to enhance the brain glioma CDT effect, and CDT monitoring was carried out by in vivo MRI [12]. In addition, it is worth noting that Mn_3_O_4_ contains Mn^2+^ and Mn^3+^. It has been reported that Mn_3_O_4_ is highly sensitive to the redox environment in the cell and is easily decomposed when exposed to GSH. The Mn^2+^ generated after the decomposition of Mn_3_O_4_ enhances T1-weighted MRI [27]. Therefore, it is reasonable to speculate that magnetic Mn_3_O_4_ can not only achieve tumor self-enhanced CDT but can also be used as a T1-enhanced MRI contrast agent. Last but not least, studies have shown that Mn_3_O_4_ has wide absorption in the near-infrared region and can be used as a new type of inorganic photothermal conversion nanoagent for photothermal therapy (PTT) of tumors [28]. Meanwhile, PTT increases the efficiency of the Fenton reaction and the productivity of ·OH by heating the tumor locally, thereby overcoming the problem of poor CDT effect caused by the limited content of endogenous H_2_O_2_ and GSH in tumor cells [13]. Therefore, the combination of CDT and PTT based on Mn_3_O_4_ as a contrast agent undoubtedly provides a reliable choice for the treatment of MDR gastric cancer. However, there is no related research on CDT/PTT synergistic therapy for MDR gastric cancer based on Mn_3_O_4_. In view of this, the establishment of a multifunctional self-enhanced nanoplatform based on Mn_3_O_4_ NPs plays a very important role in the accurate monitoring and individualized treatment of MDR in gastric cancer.

Here, we report a GMBP1-modified pH/H_2_O_2_/GSH-responsive Mn_3_O_4_ multifunctional self-enhanced nanoplatform with Fenton-like Mn^2+^ delivery, GSH depletion properties and photothermal conversion properties for in vivo real-time monitoring of MDR and in situ CDT/PTT synergistic therapy, which provides a good nanoplatform for the clinical treatment of MDR gastric cancer. Multifunctional Mn_3_O_4_ NPs were coated with polydopamine (PDA) to form Mn_3_O_4_@PDA NPs to improve their biocompatibility and enhance the effect of PTT [29]. At the same time, to achieve accurate monitoring of MDR in gastric cancer, GMBP1 was coupled to the surface of Mn_3_O_4_@PDA NPs through photoclick chemistry, and multifunctional composite MPG NPs were prepared. After MPG NPs are internalized into MDR gastric cancer cells, Mn_3_O_4_ NPs will undergo a redox reaction with GSH in the cells to generate Mn^2+^ with excellent Fenton-like activity, thereby converting endogenous H_2_O_2_ into highly toxic ·OH. The consumption of the antioxidant GSH in the cell prevents the elimination of ·OH, which leads to the enhancement of CDT. At the same time, the undissociated Mn_3_O_4_@PDA NPs produce a photothermal effect triggered by the 808 nm NIR laser, which enhances the CDT effect. In addition, Mn^2+^ with higher longitudinal relaxation properties gives Mn_3_O_4_ NPs an enhanced MRI contrast effect activated by pH/H_2_O_2_/GSH. MPG NPs will provide a new nanoplatform for the accurate monitoring and individualized treatment of MDR in gastric cancer.

## Results and discussion

**Fig. 1:**
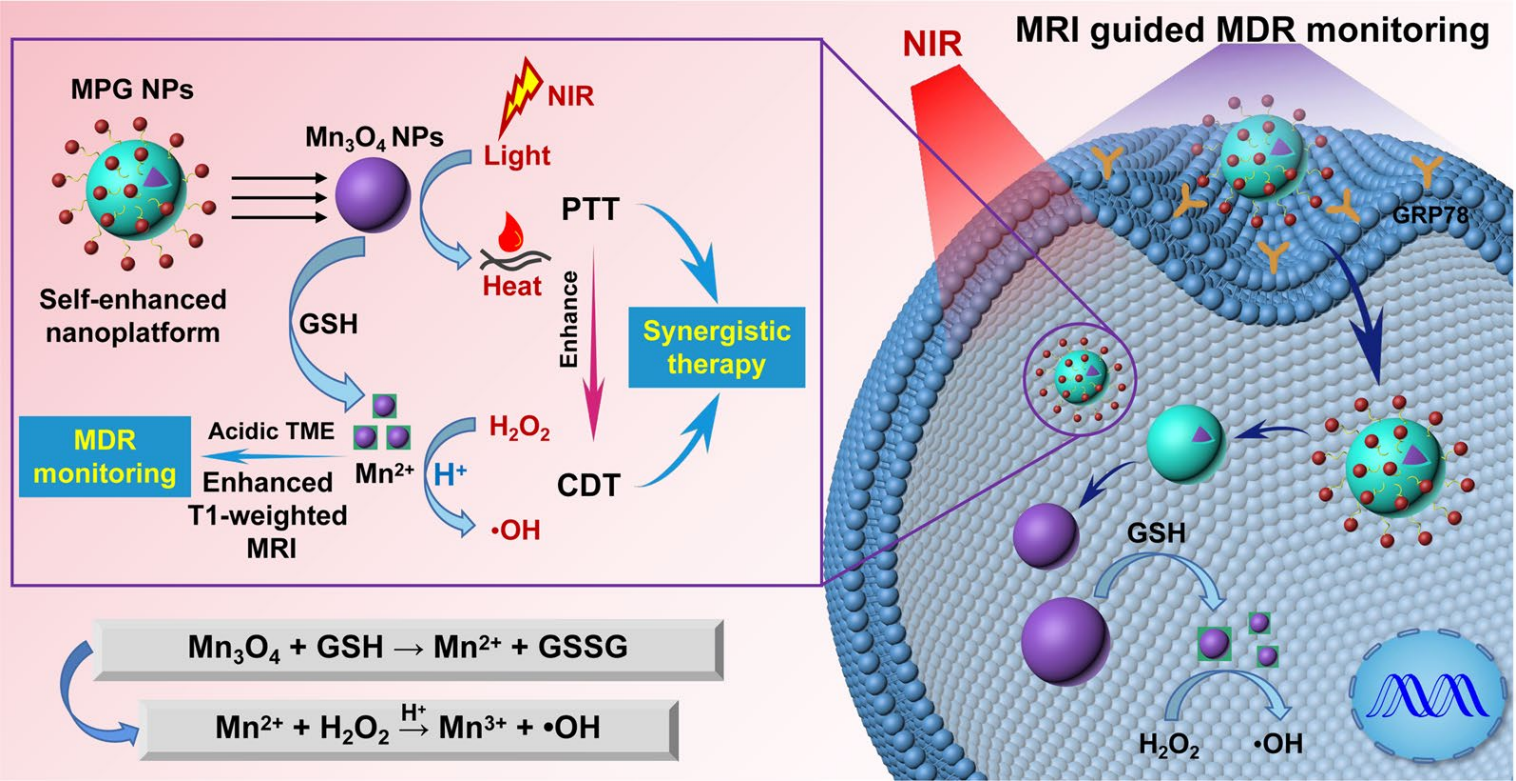
The mechanism of pH/H_2_O_2_/GSH-responsive MPG NPs as a multifunctional self-enhanced nanoplatform for gastric cancer MDR monitoring and CDT/PTT synergistic therapy. After endocytosis, MPG NPs can react with intracellular GSH by redox reaction to produce Mn^2+^, which has Fenton-like activity and can convert endogenous H_2_O_2_ into highly toxic ·OH under HCO_3_^−^ conditions. Mn^2+^ can enhance MRI for in vivo MDR monitoring. Under laser irradiation, MPG NPs can perform CDT/PTT synergistic therapy of MDR in gastric cancer

Accurate monitoring of MDR is very important for individualized treatment of gastric cancer. There have been methods to assess MDR in past studies, such as fresh tumor cell culture tests, cancer biomarker tests, or in vivo tumor imaging to assess the degree of MDR. However, to a certain extent, these methods cannot reflect the real state of MDR in real time. Therefore, it is necessary to explore new methods for real-time monitoring of MDR. Once MDR is monitored, individualized treatment can be performed on MDR gastric cancer patients to improve their survival rate. In view of this, a GMBP1 cross-linked self-enhanced nanoplatform of Mn_3_O_4_ NPs with a GSH consumption function was established, which can react with GSH to generate Mn^2+^ to enhance T1-weighted MRI, thereby mediating in vivo MDR monitoring. As a Fenton-like reagent, Mn^2+^ can convert H_2_O_2_ in cells into highly toxic ·OH, which can exert a CDT effect. Mn_3_O_4_ NPs can also be used as photothermal conversion agents for PTT, which can effectively improve Fenton reaction efficiency and the production of ·OH, thereby enhancing the CDT effect. MPG NPs achieved in vivo CDT/PTT synergistic therapy, providing a good nanoplatform for the treatment of MDR gastric cancer (Fig. 1).

### Preparation and characterization of MPG NPs

**Fig. 2:**
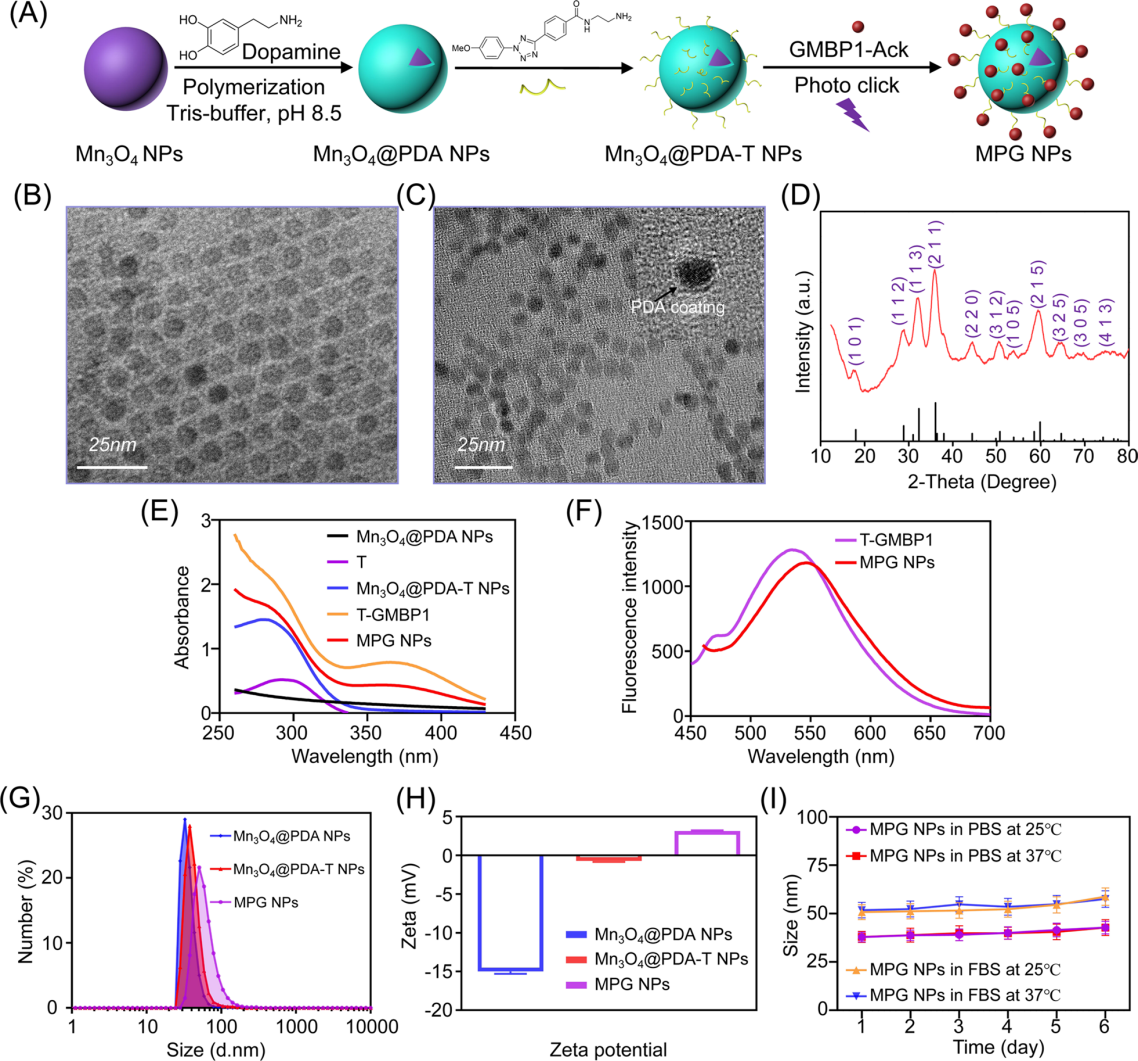
Characterization of MPG NPs. **A** Synthesis of MPG NPs. **B** TEM of Mn_3_O_4_ NPs. **C** TEM of Mn_3_O_4_@PDA NPs. **D** XRD spectrum of Mn_3_O_4_ NPs. **E** UV absorption spectra of Mn_3_O_4_@PDA NPs, T, Mn_3_O_4_@PDA-T NPs, T-GMBP1, and MPG NPs. **F** Fluorescence spectrum of T-GMBP1 and MPG NPs. **G** The size of Mn_3_O_4_@PDA NPs, Mn_3_O_4_@PDA-T NPs and MPG NPs. **H** The zeta potential of Mn_3_O_4_@PDA NPs, Mn_3_O_4_@PDA-T NPs and MPG NPs. **I** The size change of MPG NPs in different solvents at different temperatures

The synthesis scheme of MPG NPs is shown in Fig. 2A. Among them, Mn_3_O_4_ NPs were synthesized according to the thermal decomposition method described in a previous study [30]. As shown in the TEM image of Fig. 2B, the Mn_3_O_4_ NPs dispersed in the organic solvent present a spherical structure with uniform particle size, good dispersibility, and a particle size of approximately 7.4 ± 0.21 nm. The Mn_3_O_4_ NPs can be well dispersed in water after being coated with PDA. The particle size of Mn_3_O_4_ NPs coated with the PDA layer is slightly larger, approximately 8.2 ± 0.27 nm, and the PDA coating on the surface can be observed clearly (illustration) (Fig. 2C). The crystal form of Mn_3_O_4_ NPs was verified by XRD (Fig. 2D). The results show that the diffraction peaks of the synthesized Mn_3_O_4_ NPs are basically coincident with the diffraction peaks of Joint Committee on Powder Diffraction Standards (JCPDS) Card No. 24–0734, which belong to the tetragonal system. The synthetic method of tetrazole compound T is shown in Additional file 1: Fig. S1A. To verify the successful synthesis of T, the mass spectrum (Additional file 1: Fig. S1B) and ^13^ C NMR spectrum (Additional file 1: Fig. S1C) of T were obtained. ESI-MS: *m/z* 339.1 [M + H]^+^; ^13^ C NMR (150 MHz, DMSO-*d*_*6*_) δ = 38.45, 38.59, 55.06, 114.47, 121.08, 125.75, 135.89, 159.83, 163.05, 164.19, 164.98. The amino group of T and the oxidation state quinone on the surface of PDA form a covalent graft through the Schiff base reaction. The reaction easily proceeds without complicated equipment and harsh conditions [31]. As shown in the ultraviolet absorption spectrum of Fig. 2E, tetrazole compound T has a maximum absorption peak at 290 nm. After the photoclick reaction between T and GMBP1-Ack, there is a maximum absorption peak at approximately 370 nm. This is due to the formation of new pyrazoline products after the photoclick reaction [32]. Mn_3_O_4_@PDA NPs have no characteristic peak of ultraviolet absorption. Mn_3_O_4_@PDA-T NPs had the maximum absorption peak at approximately 285 nm, which proved the successful crosslinking of T. After the photoclick reaction between Mn_3_O_4_@PDA-T and GMBP1-Ack, there was a maximum absorption peak at approximately 365 nm. The blueshift of the absorption of Mn_3_O_4_@PDA-T NPs may be caused by the steric hindrance of PDA. These results initially proved the successful synthesis of MPG NPs. As shown in the fluorescence spectrum of Fig. 2F, under 410 nm laser excitation, the maximum emission wavelength after the photoclick reaction of T and GMBP1-Ack is approximately 535 nm, and the maximum emission wavelength of MPG NPs is approximately 545 nm. This is due to the fluorescent pyrazoline product produced by the photoclick reaction. As shown in Fig. 2, G and H, the hydrated particle size and zeta potential of Mn_3_O_4_@PDA NPs, Mn_3_O_4_@PDA-T NPs and MPG NPs were measured by a Malvern particle size analyzer. The results show that the hydrated particle size of Mn_3_O_4_@PDA NPs is approximately 32.7 ± 4.8 nm, and the potential is −15 mV. The negative potential of Mn_3_O_4_@PDA NPs is attributed to the quinoneimine and catechol groups on the surface of PDA. The reversible dissociation and deprotonation/protonation of the amine and catechol groups make PDA generate a positive or negative charge, and the net charge is negative [33]. After crosslinking T on the surface of Mn_3_O_4_@PDA NPs, the hydrated particle size increases to approximately 37.8 ± 5.6 nm, and the potential tends to be neutral. After the photoclick reaction between Mn_3_O_4_@PDA-T NPs and GMBP1-Ack, the particle size continues to increase to approximately 50.5 ± 3.7 nm, which is due to the influence of the surface hydration layer, and the potential has also become + 3 mV. The stability of nanoparticles is an important factor for in vivo long-term imaging studies. The MPG NPs were dispersed in PBS and 10% FBS, and the changes in particle size at different temperatures were measured to verify the stability of the MPG NPs. As shown in Fig. 2I, the particle size of MPG NPs did not change significantly within 6 days, and the solution remained clear. The results indicate that MPG NPs have good stability.

### In vitro MRI and fluorescence imaging of MPG NPs

**Fig. 3:**
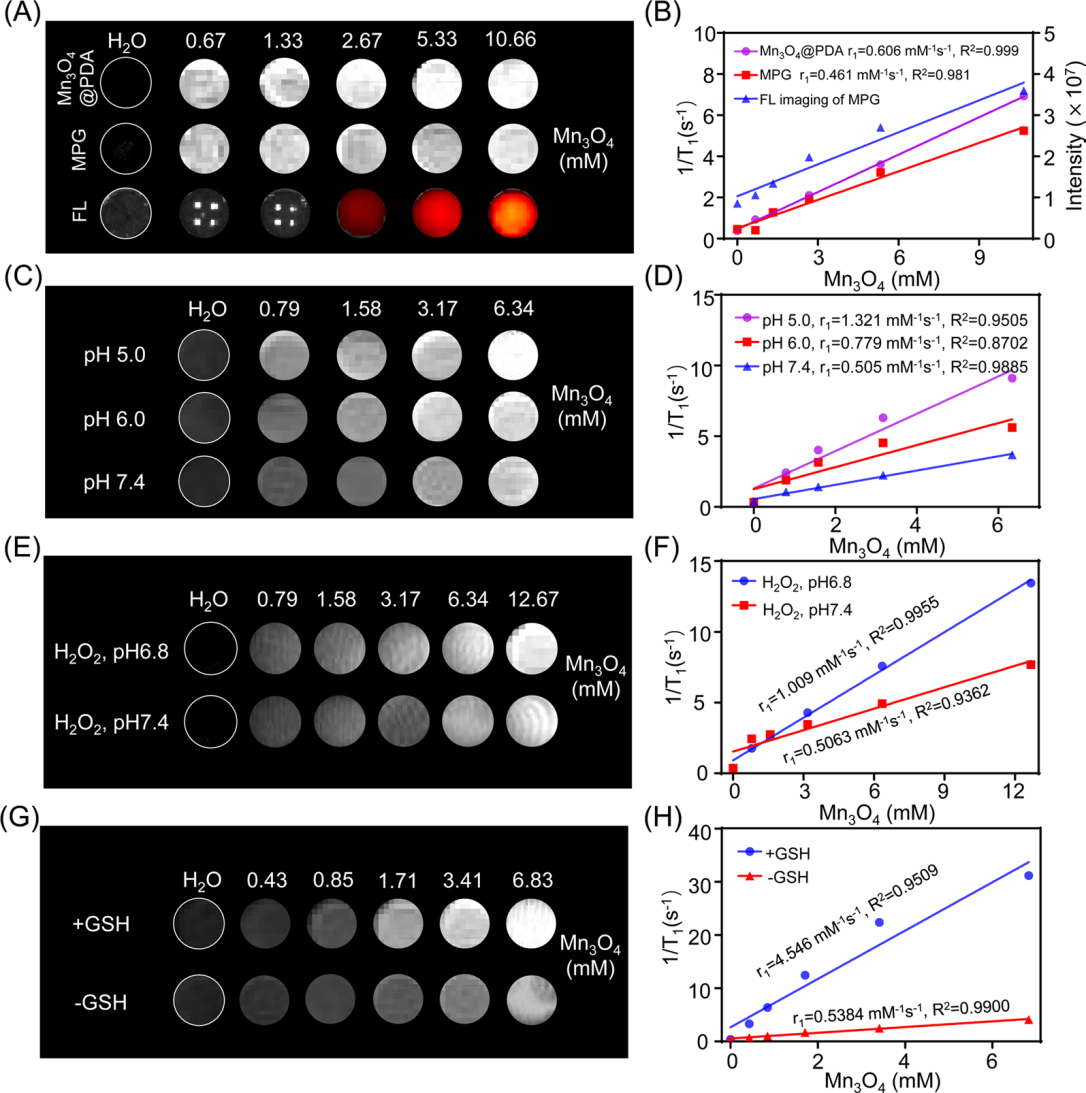
In vitro MRI and fluorescence imaging of MPG NPs. **A** MRI and fluorescence imaging of different concentrations of Mn_3_O_4_@PDA NPs and MPG NPs. **B** MRI relaxation rate and fluorescence intensity of Mn_3_O_4_@PDA NPs and MPG NPs at different concentrations. **C** MRI of different concentrations of MPG NPs at different pH values. **D** MRI relaxation rate of MPG NPs at different concentrations and different pH values. **E** MRI of different concentrations of MPG NPs with H_2_O_2_ at different pH values. **F** MRI relaxation rate of MPG NPs with H_2_O_2_ at different concentrations and different pH values. **G** MRI of different concentrations of MPG NPs with or without GSH. **H** MRI relaxation rate of MPG NPs with or without GSH at different concentrations

To test the MRI contrast ability of MPG NPs, the relaxation characteristics of Mn_3_O_4_@PDA NPs and MPG NPs were measured with MRI scanner (0.5 T). As shown in Fig. 3A, as the concentration of Mn_3_O_4_ NPs increased, Mn_3_O_4_@PDA NPs and MPG NPs both showed signal enhancement in the T1-weighted MRI image. According to the linear fit of 1/T1 to the concentration of Mn_3_O_4_ NPs, the r1 values of Mn_3_O_4_@PDA NPs and MPG NPs were calculated to be 0.606 mM^− 1^s^− 1^ and 0.461 mM^− 1^s^− 1^, respectively (Fig. 3B). PDA modification enhances the T1 relaxivity of Mn_3_O_4_ NPs, and its r1 value is more than 3 times higher than that of Mn_3_O_4_@PEG NPs (0.2 mM^− 1^s^− 1^) [7], confirming the potential use of Mn_3_O_4_@PDA NPs as MRI imaging agents. The r1 value of MPG NPs is lower than that of Mn_3_O_4_@PDA NPs. The main reason for this result is that the connection of T on the surface of PDA reduces the contact of water protons with Mn_3_O_4_ nuclei, thereby weakening the T1 relaxation properties of NPs. Although the r1 value of MPG NPs is reduced, it is still higher than that of Mn_3_O_4_@PEG NPs, indicating that it has the ability for in vivo MRI. At the same time, the in vitro fluorescence imaging performance of MPG NPs was tested by the PerkinElmer IVIS system. As shown in Fig. 3A, as the concentration of MPG NPs increased, the fluorescence signal increased, which was consistent with the result of fluorescence signal extraction in the region of interest (Fig. 3B), indicating that GMBP1 successfully crosslinked with Mn_3_O_4_@PDA NPs through the photoclick reaction.

Previous studies have shown that pH or GSH can enhance T1-weighted MRI of Mn_3_O_4_ NPs [27, 34, 35] because Mn_3_O_4_ can generate Mn^2+^ in response to the TME. To verify whether MPG NPs can respond to the TME, the pH sensitivity of MPG NPs was first verified. During tumor growth, glycolysis and metabolism are upregulated to produce a large amount of lactic acid, resulting in a lower pH in tumor tissues and weak acidity of the TME [36]. To simulate the blood environment and TME, the pH values were set to 7.4, 6.0 and 5.0 [37]. The relaxation images and relaxation times of MPG NPs at different pH values were measured, and the relaxation rate was calculated. As shown in Fig. 3C, under the same pH conditions, with increasing Mn_3_O_4_ concentration in MPG NPs, the MRI signal gradually increased. At the same Mn_3_O_4_ concentration, the lower the pH value, the stronger the MRI signal. Figure 3D shows the relaxation rate at different pH values. The results show that the relaxation rate r1 is only 0.505 mM^− 1^s^− 1^ at pH 7.4 and 0.779 mM^− 1^s^− 1^ at pH 6.0. When the pH value is 5.0, the relaxation rate can reach 1.321 mM^− 1^s^− 1^, which is 2.6 times that under neutral conditions. These results indicated that the synthesized MPG NPs are sufficiently sensitive to acidic environments. Under acidic conditions, the MRI signal increases significantly, the relaxation time becomes shorter, and the relaxation rate increases. Furthermore, MPG NPs can be used as excellent pH-responsive T1-weighted MRI agents.

Furthermore, H_2_O_2_ is overproduced in malignant tumor cells, leading to a significant increase in H_2_O_2_ in the TME [38]. There have been reports of H_2_O_2_ dissociating MnO_2_ into O_2_ and Mn^2+^ under weak acid conditions to enhance T1-weighted MRI [39, 40], but there is no report in terms of Mn_3_O_4_-related research. Therefore, the T1-MRI performance of Mn_3_O_4_ under H_2_O_2_ conditions was explored. In Fig. 3E, it is observed that MPG NPs have strong concentration-dependent signal enhancement. In addition, the relaxation rate of MPG NPs at pH 6.8 (r1 = 1.009 mM^− 1^s^− 1^) is 1.9 times that at pH 7.4 (r1 = 0.5063 mM^− 1^s^− 1^) (Fig. 3F), indicating that protons are essential in the decomposition of Mn_3_O_4_ induced by H_2_O_2_. At the same time, after adding H_2_O_2_, the T1 enhancement obtained in the presence of H^+^ is also attributed to the catalytic reaction of H_2_O_2_ reduction [41, 42].

In addition, GSH in tumor tissue is 5 times that of normal tissue, and GSH plays a key role in protecting cells from various harmful substances (such as H_2_O_2_, superoxide, ·OH and other active substances) [12]. To evaluate the effectiveness of MPG NPs as GSH-activated T1 contrast agents, MPG NPs were dispersed in a solution containing GSH (15 mM) and incubated for 5 min, and T1-weighted MRI images were obtained (Fig. 3G). In the presence of GSH, T1-weighted MRI images of MPG NPs showed stronger signal enhancement than MPG NP solutions without GSH, and the r1 relaxation rate was increased by 7.4 times (Fig. 3H). The enhancement of the relaxation rate is due to the release of Mn^2+^ after the redox reaction between Mn_3_O_4_ NPs and GSH, and the water coordination number in the center of Mn increases, which leads to the enhancement of the T1-weighted MRI signal [27]. The above results proved that MPG NPs have the potential to respond to TME and enhance MRI, which can be used as good self-enhanced nanoplatforms.

### In vitro chemodynamic activity of MPG NPs

**Fig. 4:**
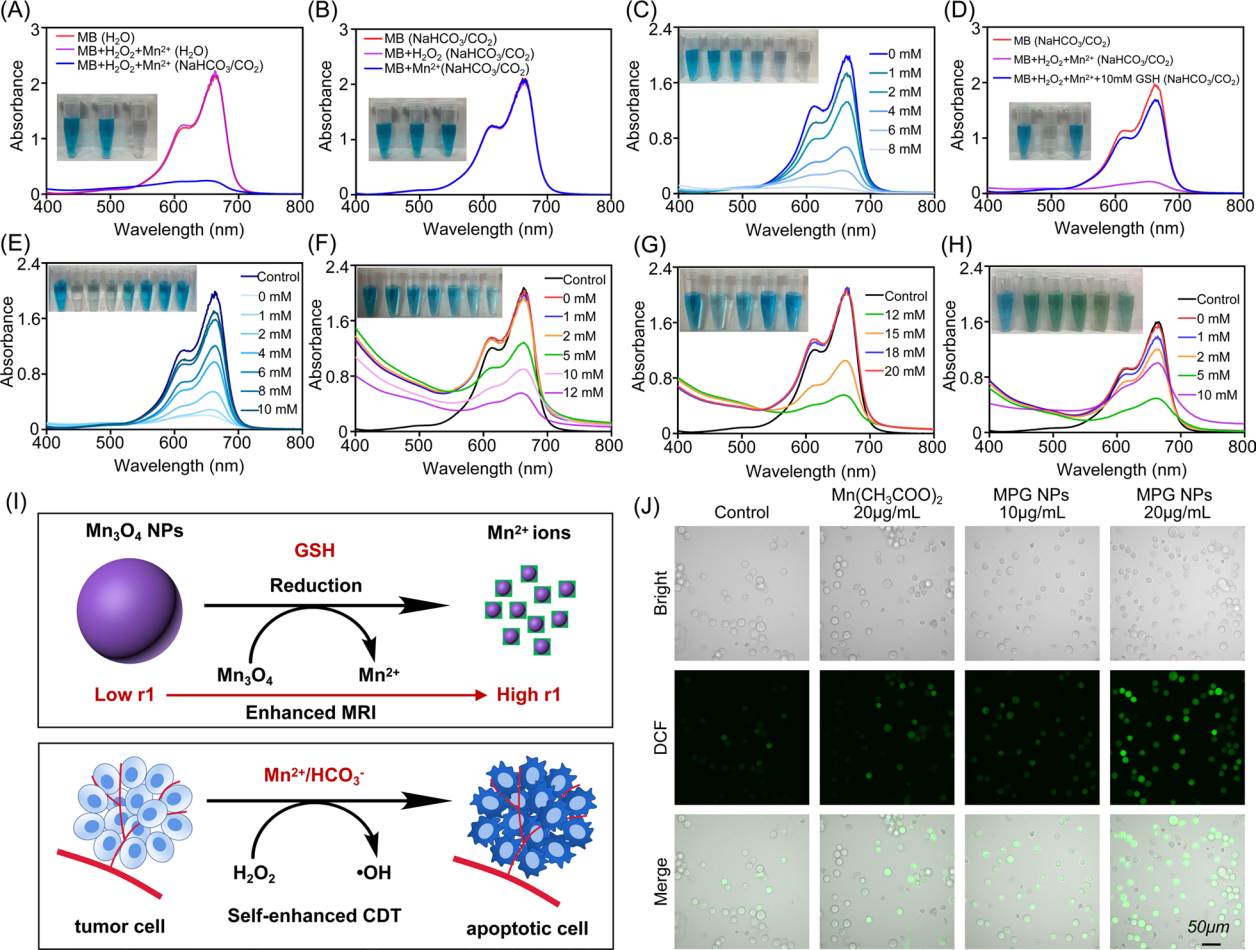
In vitro chemodynamic activity of MPG NPs. **A** UV-visible absorption spectra and photo (inset) of MB degradation induced by Mn^2+^ in different solutions. **B** UV-visible absorption spectra and photo (inset) of MB in the presence of Mn^2+^ and H_2_O_2_ alone. **C** UV-visible absorption spectra and photo (inset) of MB degradation induced by Mn^2+^ when the concentration of H_2_O_2_ gradually increases. **D** UV-visible absorption spectra and photo (inset) of MB degradation induced by Mn^2+^ with or without GSH. **E** UV-visible absorption spectra and photo (inset) of MB degradation induced by Mn^2+^ when the concentration of GSH gradually increases. **F** UV-visible absorption spectra and photo (inset) of MB degradation induced by Mn^2+^ (Mn_3_O_4_@PEG) when the concentration of GSH gradually increases. **G** UV-visible absorption spectra and photo (inset) of MB degradation induced by Mn^2+^ (Mn_3_O_4_@PEG) when the concentration of GSH gradually increases. **H** UV-visible absorption spectra and photo (inset) of MB degradation induced by Mn^2+^ (MPG NPs) when the concentration of GSH gradually increases. **I** Schematic diagram of MPG NPs reacting with GSH to produce Mn^2+^ and converting endogenous H_2_O_2_ into ·OH. **J** DCF fluorescence of SGC 7901 ADR with or without MPG NPs

The occurrence of MDR is closely related to TME. To verify whether MPG NPs can respond to the TME and exert in vivo CDT effect, the chemodynamic activity of MPG NPs was studied in vitro. First, it was verified that Mn^2+^ can react with H_2_O_2_ to produce ·OH through a Fenton-like reaction in the presence of HCO_3_^−^, thereby degrading methylene blue (MB). It can be seen from Fig. 4A and B that when Mn^2+^ and H_2_O_2_ are incubated in NaHCO_3_/CO_2_ buffer for 30 min, the absorbance of MB at 665 nm is significantly reduced, and the presence of Mn^2+^ or H_2_O_2_ alone has no significant effect on the absorbance of MB. In aqueous solution, the absorbance value of MB did not change significantly. As the concentration of H_2_O_2_ increased, the absorbance of MB gradually decreased (Fig. 4C). These results show that the Mn^2+^-mediated Fenton-like reaction can effectively produce ·OH under physiological NaHCO_3_/CO_2_ conditions, which is consistent with previous research [12]. In Fig. 4D, in the presence of 10 mM GSH, the absorbance value of MB did not significantly decrease, which indicates that ·OH-induced MB degradation was significantly inhibited in the presence of 10 mM GSH. With increasing GSH concentration (0–10 mM), the absorbance value of MB gradually increased, indicating that the degradation of MB was gradually inhibited (Fig. 4E). The remaining amount of MB in each group is shown in Additional file 1: Fig. S2A. Next, the chemodynamic activity of Mn_3_O_4_@PEG NPs was verified. In previous studies, the color of the MnO_2_ solution gradually disappeared after gradually adding different concentrations of GSH solution, and it was proven that MnO_2_ reacted with GSH to produce Mn^2+^ and GSSG. Different concentrations of GSH solution were gradually added to the Mn_3_O_4_@PEG NP solution, and the color of the solution gradually became lighter (Additional file 1: Fig. S2B), and the reaction efficiency with GSH was significantly higher than that of pH-triggered reactions (Additional file 1: Fig. S2C), which is consistent with a previous study [12]. Figure 4F shows that as the concentration of GSH in the Mn_3_O_4_@PEG NP solution increases, the absorbance value of MB gradually decreases. When the concentration of GSH reaches 12 mM, the absorbance value of MB gradually increases, indicating that Mn_3_O_4_ NPs react with GSH to produce Mn^2+^, which reacts with H_2_O_2_ to produce ·OH through a Fenton-like reaction. However, with the increase in free GSH, the ·OH that produced by the Fenton-like reaction is gradually eliminated, thereby inhibiting the degradation of MB (Fig. 4G). Figure 4H shows the degradation of MB after adding different concentrations of GSH to the MPG NP solution. The results show that as the concentration of GSH increases, the absorbance of MB decreases first and then increases gradually, which is consistent with the above results [12]. The degradation efficiency of MB by ·OH in the presence of GSH is shown in Additional file 1: Fig. S2, D to F. The results show that when the GSH concentration was as high as 10 mM, Mn_3_O_4_@PEG NPs still showed 57.69% MB degradation efficiency, which is approximately 3 times the MB degradation efficiency promoted by Mn^2+^ (18.8%) under the same conditions. MPG NPs showed 39.92% MB degradation efficiency, which is approximately 2.12 times the MB degradation efficiency promoted by Mn^2+^ under the same conditions. These results indicate that MPG NPs have good in vitro chemodynamic activity and have the potential for in vivo CDT.

The above experiments have proven that Mn_3_O_4_ NPs can generate Mn^2+^ in the presence of GSH and enhance MRI. Mn^2+^ can convert H_2_O_2_ into highly toxic ·OH to kill tumor cells (Fig. 4I). The production of ROS in the presence of MPG NPs was verified in the cell. Figure 4J shows the observation of ROS production by detecting cell DCF fluorescence after adding different materials. DCFH-DA has no fluorescence and is hydrolyzed into DCFH after entering cells and then rapidly oxidized to DCF with fluorescence [43]. The fluorescence intensity of DCF shows that in the presence of manganese ions, the fluorescence of DCF is significantly stronger than that of the control group. The quantitative analysis of the mean fluorescence intensity (MFI) is shown in Additional file 1: Fig. S2G. The results showed that the DCF fluorescence intensity of cells coincubated with MPG NPs was significantly different from that of the other groups. This indicates that MPG NPs react with GSH in cells to generate Mn^2+^, which mediates the Fenton-like reaction and generates ROS. It not only reduces the elimination of ·OH by GSH but also produces more ROS and enhances the killing effect on tumor cells.

### In vitro and in vivo photothermal effect of MPG NPs

**Fig. 5:**
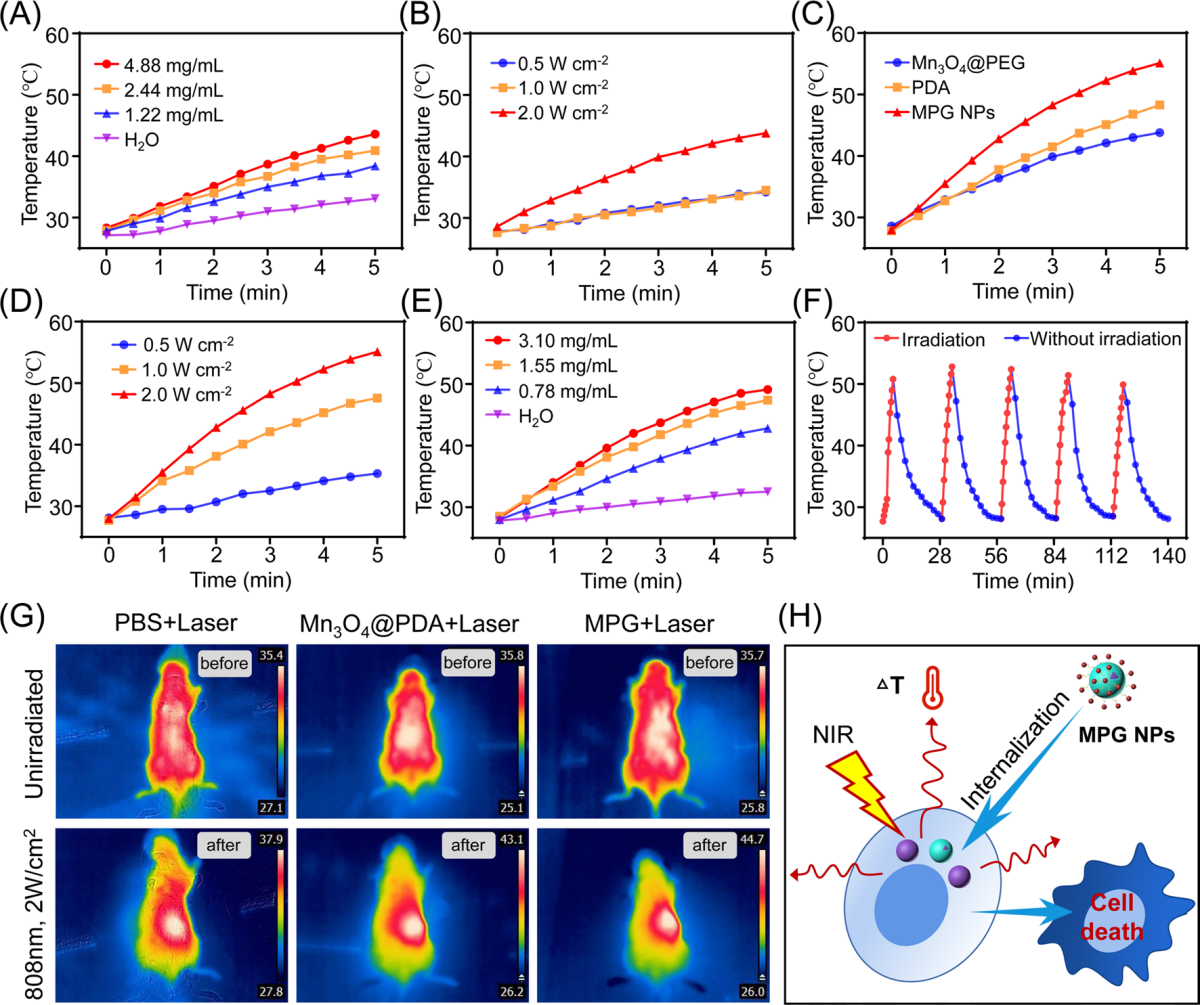
In vitro and in vivo photothermal effect of MPG NPs. **A** Temperature changes of different concentrations of Mn_3_O_4_@PEG after 5 min of laser irradiation. **B** Temperature change of Mn_3_O_4_@PEG after 5 min of laser irradiation with different powers. **C** Temperature change of the same concentration of Mn_3_O_4_@PEG, PDA and MPG NPs after 5 min of laser irradiation. **D** Temperature change of MPG NPs after 5 min of laser irradiation with different powers. **E** Temperature change of different concentrations of MPG NPs after 5 min of laser irradiation. **F** Temperature change of MPG with or without laser irradiation. **G** Temperature changes under laser irradiation after 6 h of in vivo injection of MPG NPs. **H** Schematic diagram of the photothermal effect of MPG NPs under laser irradiation after endocytosis

Studies have shown that Mn_3_O_4_ NPs have high molar extinction coefficient and strong absorption in the near-infrared region [28, 44], which can be used for in vivo PTT. In addition, PTT can increase the efficiency of the Fenton reaction and the productivity of ·OH by locally heating the tumor [13]. To verify whether MPG NPs can be used for in vivo PTT, the photothermal effect of MPG NPs was verified. First, it was determined whether Mn_3_O_4_ NPs have photothermal effects and enhanced in vivo PTT. The temperature changes of different concentrations of Mn_3_O_4_@PEG NPs irradiated with 808 nm lasers with different powers for 5 min were measured. As shown in Fig. 5A, under laser irradiation (1 W), the temperature of the aqueous solution does not change much. As the Mn_3_O_4_@PEG NP concentration increases, the temperature gradually increases. As shown in Fig. 5B, as the laser power increases, the temperature of the Mn_3_O_4_@PEG NP solution (1 mg mL^− 1^) gradually increases. These results indicate that Mn_3_O_4_@PEG NPs have certain photothermal properties. As shown in Fig. 5C, the temperature change of the solution of the same concentration of Mn_3_O_4_@PEG NPs, PDA and MPG NPs (1 mg mL^− 1^) was measured after 808 nm laser irradiation (2 W) for 5 min. The results show that the temperature of MPG NPs increases the fastest, and the temperature can reach 55 °C after 5 min. Compared with Mn_3_O_4_@PEG NPs and PDA, MPG NPs have better photothermal performance. To detect the influence of laser power on the photothermal effect of MPG NPs, the temperature change of MPG NPs under different laser powers for 5 min was measured. As shown in Fig. 5D, when the laser power is 0.5 W, the temperature of the MPG NP solution does not change much. As the power increases, the temperature gradually increases. When the power reaches 2 W, the temperature of the MPG NP solution can reach 55 °C after 5 min of irradiation. To verify the influence of the concentration on the photothermal effect of MPG NPs, the temperature changes of MPG NP solutions of different concentrations under 808 nm laser irradiation (1 W) for 5 min were measured. The solution without MPG NPs was used as a control. As shown in Fig. 5E, after 5 min of laser irradiation, the temperature of the control solution did not change much, and the temperature gradually increased as the concentration increased. To verify whether MPG NPs have good photothermal cycling properties, the MPG NP solution was first irradiated with a laser (2 W) for 5 min, and then irradiation was stopped and returned to room temperature. The cycle was repeated 5 times. As shown in Fig. 5F, the temperature of the MPG NP solution can rise above 50 °C after each irradiation and return to room temperature within a certain period of time. The results show that laser irradiation does not affect the photothermal performance of MPG NPs.

To verify the in vivo photothermal effect of MPG NPs, photothermal images of the tumor-bearing mice before and after laser irradiation were obtained. Figure 5G shows that the temperature of the tumor site of the mice in the PBS injection group did not increase significantly after laser irradiation and only increased from 35.4 to 37.9 °C. The tumor site temperature of mice injected with Mn_3_O_4_@PDA NPs increased from 35.8 to 43.1 °C. The fastest increase in temperature was observed in the mice injected with MPG NPs. The temperature of the tumor site increased from 35.7 to 44.7 °C, which also proved that MPG NPs demonstrate good targeting of MDR gastric cancer cells, allowing more MPG NPs to gather at the tumor sites to exert the better PTT effect. All of the above results indicate that MPG NPs have a good in vitro and in vivo photothermal effect and can release heat under laser irradiation after being internalized by the cells, thus causing cell death (Fig. 5H).

### Cellular uptake and affinity analysis

To verify whether MPG NPs can be used for in vivo MDR monitoring, cell uptake and affinity analyses were first performed. SGC 7901 ADR cells were used for internalization analysis, and the cell affinity of MPG NPs was verified in SGC 7901 ADR and SGC 7901 cell lines. The same concentration of MPG NPs was incubated with SGC 7901 ADR cells for different times, and the fluorescence intensity was observed by confocal microscopy. As shown in Additional file 1: Fig. S3A, blue represents the fluorescence of DAPI, and green represents the fluorescence of MPG NPs. The results showed that with increasing incubation time, the green fluorescence gradually increased, and the green fluorescence was mainly distributed in the cytoplasm, which indicated that MPG NPs were gradually internalized into the cytoplasm. The quantification of fluorescence intensity in Additional file 1: Fig. S3C also shows the same result. A cell affinity experiment was used to verify the specific targeting ability of MPG NPs on MDR gastric cancer cells. As shown in Additional file 1: Fig. S3B, in SGC 7901 ADR cells, the green fluorescence intensity after blocking with GMBP1 was significantly lower than the fluorescence intensity after MPG NP incubation. In SGC 7901 cells, the green fluorescence intensity after blocking with GMBP1 was not significantly different from the fluorescence intensity directly incubated with MPG NPs. The above results indicate that GMBP1 has the ability to specifically target SGC 7901 ADR cells because GMBP1 can specifically bind to the GRP78 receptor overexpressed on the surface of MDR cells [5]. The quantitative analysis shown in Additional file 1: Fig. S3D also produced the same results. The good specific targeting of MPG NPs to tumor cells also leads to less accumulation of MPG NPs in normal cells, thereby further reducing its in vivo toxicity. MPG NPs provide the basis for in vivo MDR monitoring and synergistic therapy with their specific tumor targeting.

### Cellular inhibitory effect of MPG NPs

**Fig. 6:**
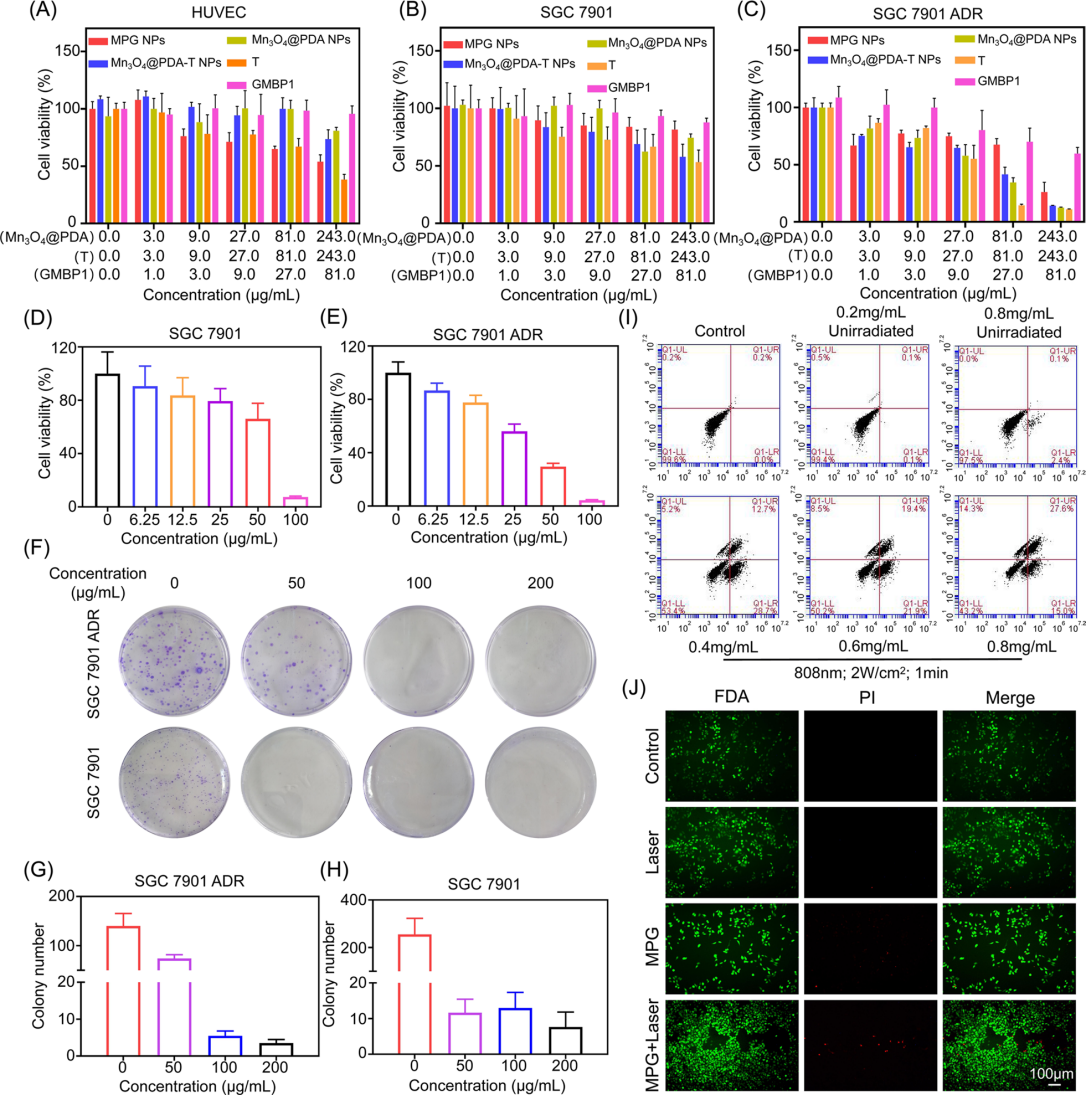
Tumor cell inhibitory effect of MPG NPs. **A** Survival rate of HUVECs after incubation with MPG NPs. **B** Survival rate of SGC 7901 cells after incubation with MPG NPs. **C** Survival rate of SGC 7901 ADR cells after incubation with MPG NPs. **D** Survival rate of SGC 7901 cells incubated with MPG NPs after laser irradiation. **E** Survival rate of SGC 7901 ADR cells incubated with MPG NPs after laser irradiation. **F** Clone images of cells incubated with MPG NPs after laser irradiation. **G** Quantification of the number of clones in SGC 7901 ADR cells. **H** Quantification of the number of clones in SGC 7901 cells. **I** Flow cytometry of cells incubated with different concentrations of MPG NPs with or without laser irradiation. **J** Staining image of cells with or without laser irradiation after incubation with MPG NPs

The toxicity and the inhibitory effect of nanomaterials on normal cells and tumor cells were evaluated by the MTT method, which is very important for MDR gastric cancer therapy. As shown in Fig. 6A, the cytotoxicity of MPG NPs to normal cells was concentration-dependent. With increasing MPG NP concentration, the survival rate of HUVECs decreased gradually. In previous studies, cell survival was still close to 60% when the concentration of Mn_3_O_4_@PEG was up to 40 µg mL^− 1^ [7]. The coating of PDA significantly reduced the toxicity of Mn_3_O_4_, and when the concentration reached 243 µg mL^− 1^, the survival rate of normal cells after Mn_3_O_4_@PDA NPs treatment was still above 80%. In SGC 7901 cells, MPG NPs did not show a significant inhibitory effect on the cells (Fig. 6B), while in SGC 7901 ADR cells, MPG NPs had a significantly enhanced inhibitory effect on the cells (Fig. 6C). The results show that low concentrations of MPG NPs will not cause significant damage to normal cells, and the presence of GMBP1 reduces the cytotoxicity caused by the tetrazolium compound T. Moreover, the high uptake of MPG NPs by SGC 7901 ADR cells also increased the inhibitory effect of MPG NPs on MDR gastric cancer cells, which was attributed to the presence of GMBP1 on the surface of MPG NPs. The survival rate of the cells after laser irradiation was measured in SGC 7901 and SGC 7901 ADR cells. As shown in Fig. 6D and E, laser irradiation did not cause significant cell death in the absence of MPG NPs. With increasing MPG NP concentration, the cell survival rate decreased significantly. When the concentration of MPG NPs reached 100 µg mL^− 1^, only a few cells survived, and under laser irradiation, the inhibitory effect of MPG NPs on SGC 7901 ADR cells was significantly stronger than that of SGC 7901 cells.

The inhibitory effect of MPG NPs on tumor cells was verified by colony formation assay. Images of cell clones treated with different concentrations of MPG NPs were obtained, and the number of colonies was quantified. As shown in Fig. 6F, in SGC 7901 and SGC 7901 ADR cells, the number of cell clones in the control group was the largest. With increasing MPG NP concentration, the number of cell clones gradually decreased. Figure 6G and H show the quantification of the number of cell clones in each group. The results show that when the MPG NP concentration reaches 100 µg mL^− 1^, the number of cell clones is significantly reduced, which is a significant difference compared with the control group. The above results indicate that MPG NPs have a good photothermal effect and can inhibit the growth of tumor cells after 808 nm laser irradiation. With increasing MPG NP concentration, the photothermal effect is more obvious, and the tumor cell inhibitory effect is also more obvious.

The apoptosis of cells after incubation with different concentrations of MPG NPs was observed by flow cytometry. As shown in Fig. 6I, the cells that were not treated with MPG NPs did not show obvious apoptosis. Without laser irradiation, slight early apoptosis appeared gradually with increasing MPG NP concentration. However, after 808 nm laser irradiation for 2 min, the number of apoptotic cells increased with increasing MPG NP concentration. The results show that the photothermal effect of MPG NPs causes significant cell apoptosis, verifying the good photothermal effect and the potential of in vivo PTT of MPG NPs. The inhibitory effect of MPG NPs on SGC 7901 ADR cells was further verified by cell staining. FDA was used to stain living cells with green fluorescence, and PI was used to stain dead cells with red fluorescence. As shown in Fig. 6J, in the absence of MPG NPs, neither laser irradiation nor nonirradiation caused significant cell death. The low concentration of MPG NPs only caused a few cell deaths, which is due to the CDT effect of MPG NPs in the cells. At the same concentration of MPG NPs, the number of cell deaths increased greatly after laser irradiation, which was caused by the photothermal effect of MPG NPs after laser irradiation. All the above results indicated that MPG NPs had a significant inhibitory effect on tumor cells and had the potential for synergistic therapy in vivo.

### In vivo T1-weighted MRI for MDR monitoring

**Fig. 7:**
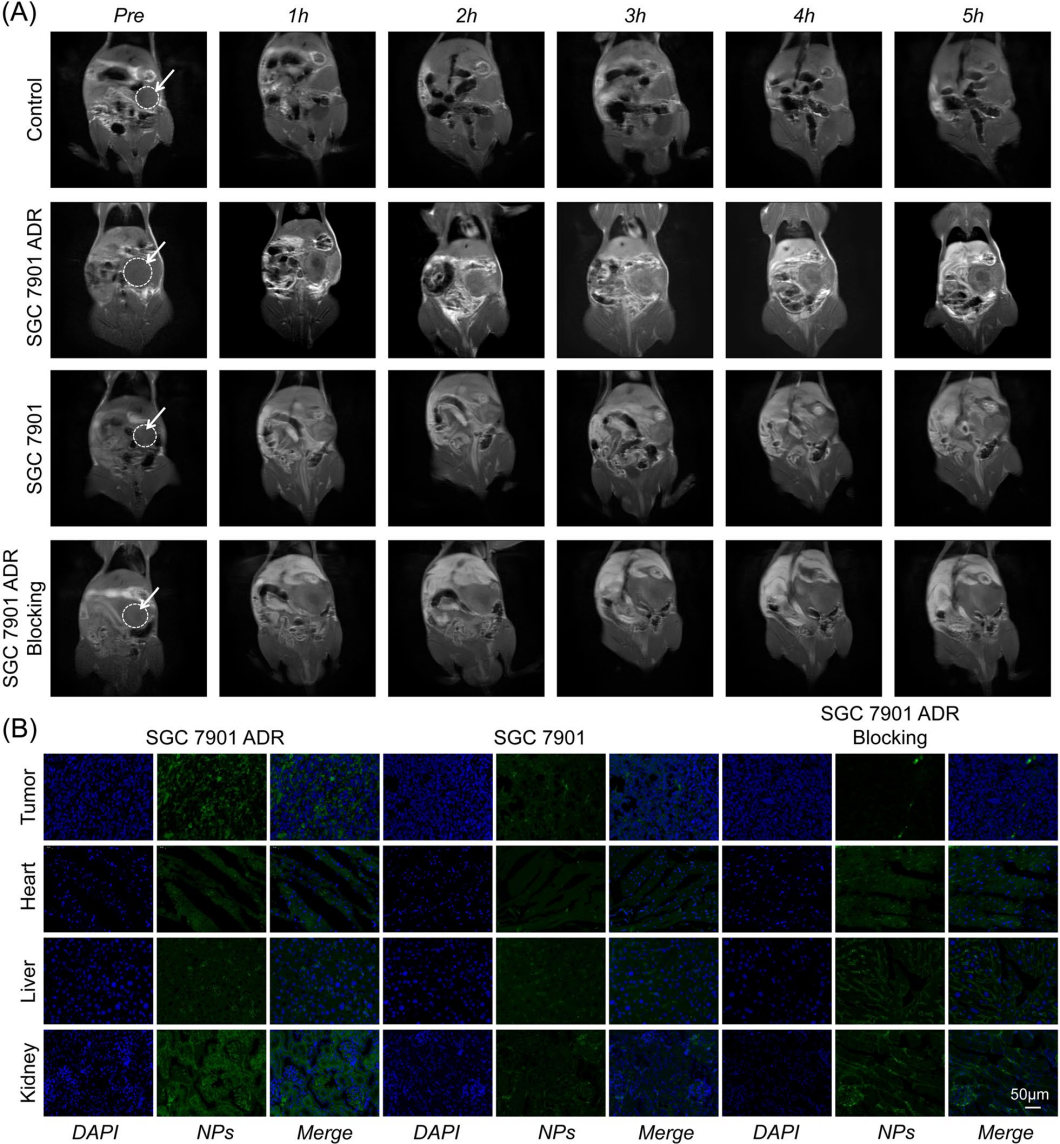
In vivo MRI and immunofluorescence staining. **A** In vivo MRI images of mice in each group. **B** Immunofluorescence staining images of the main organs and tumor tissue sections of mice in each group

In vivo MDR monitoring is very important for the individualized treatment of gastric cancer. MRI was performed to verify whether MPG NPs can be used for in vivo MDR monitoring. An orthotopic gastric cancer mouse model was established, MPG NPs were injected in vivo, and MRI signal changes were monitored for 5 h. As shown in Fig. 7A, no significant signal enhancement at the tumor site was observed in the control group. The tumor site has been marked with a circle. The MRI signal intensity of the mice in the SGC 7901 ADR group increased significantly 2 h after the injection, and the MRI signal intensity was strongest at 3 h after the injection. In the SGC 7901 group, the MRI signal intensity also increased significantly after injection, but the signal intensity was weaker than that of the SGC 7901 ADR group. In the blocking group, GMBP1 was injected first, and then MPG NPs were injected half an hour later, the MRI signal intensity did not increase significantly. This is because free GMBP1 competes with MPG NPs to bind to the GRP78 receptors on the surface of MDR gastric cancer cells, so MPG NPs cannot be enriched at the tumor site, thus showing lower MRI signal intensity. It can also be seen from the quantitative results that in SGC 7901 ADR model mice, the MRI signal increased significantly and reached the maximum signal value 3 h after injection (Additional file 1: Fig. S3E). Major organs and tumor tissues of each group of mice were obtained, and immunofluorescence staining was performed. Figure 7B shows the staining images of each group of mice. The results showed that the fluorescence intensity of MPG NPs in the SGC 7901 ADR mouse model was significantly higher than that in the SGC 7901 mouse model and GMBP1 blocking group. This was attributed to the presence of the specific ligand GMBP1 on the surface of MPG NPs, which allowed more MPG NPs to enter SGC ADR cells. This is consistent with the results of in vivo MRI. These results indicate that GMBP1 can be used as a specific peptide to monitor MDR in gastric cancer.

### In vivo synergistic therapy for orthotopic MDR gastric cancer

**Fig. 8:**
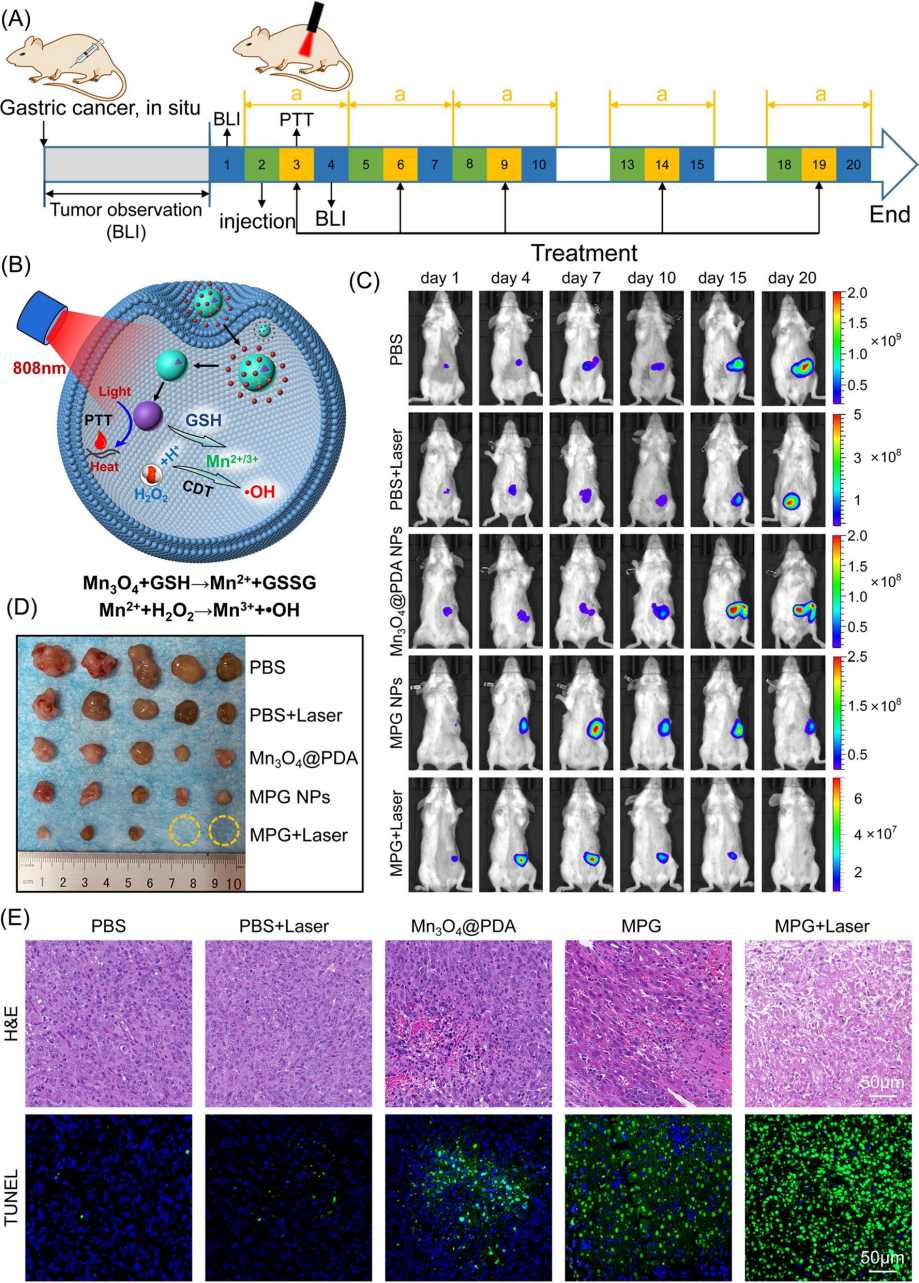
In vivo synergistic therapy. **A** synergistic therapy strategy for 20 days. **B** Schematic diagram of in vivo synergistic therapy. **C** Bioluminescence images of mice in each group within 20 days. **D** Photos of tumors in each group of mice. **E** H&E staining and TUNEL staining images of tumor sections of mice in each group

The development of MDR is closely related to the TME, and tumor treatment strategies for the TME have been widely used to combat MDR. In vivo MRI has achieved accurate monitoring of MDR. The results of in vitro experiments have proven that MPG NPs can consume GSH and convert the endogenous H_2_O_2_ of the cell into highly toxic ·OH, thereby exerting the CDT effect. The good photothermal effect of Mn_3_O_4_ NPs can realize in vivo PTT, which can further enhance the CDT effect. To verify whether MPG NPs can be used for in vivo CDT/PTT synergistic therapy, an orthotopic MDR gastric cancer mouse model was established. As shown in Fig. 8A, the tumorigenesis of model mice was monitored by bioluminescence imaging, and synergistic therapy was performed for 20 days when the tumor size increased to an appropriate size. 6 h after the injection, the tumor sites of the mice in the PTT group were irradiated with an 808 nm laser. The bioluminescence imaging images of the mice were obtained 12 h after irradiation, and the weight changes of the mice were recorded. Figure 8B shows the simple principle of synergistic therapy. MPG NPs gather at the tumor site with abundant blood vessels through the EPR effect and the specific targeting effect of GMBP1 along with the blood circulation. Under irradiation with an 808 nm laser, Mn_3_O_4_@PDA NPs convert light energy into heat energy and locally release heat, thereby causing tumor cell apoptosis. The released Mn_3_O_4_ NPs react with GSH in tumor cells to generate Mn^2+^. Mn^2+^ and H_2_O_2_ in tumor cells mediate the Fenton-like reaction under HCO_3_^−^ conditions to produce highly toxic ·OH, thereby killing tumor cells. The consumption of GSH by Mn_3_O_4_ also inhibits the elimination of ·OH by GSH to achieve a better CDT effect.

Figure 8C shows the bioluminescence images of each group of mice after 20 days of treatment. In the PBS group, the signal at the tumor site gradually increased, indicating that the tumor gradually became larger without treatment. After PBS injection and 808 nm laser irradiation, the signal at the tumor site also showed a gradual increase, but the signal was weaker than that in the PBS group. This indicates that laser irradiation alone can slow down the growth of the tumor to a certain extent, but the therapeutic effect is not better because of the low temperature. The bioluminescence signal of the mice injected with Mn_3_O_4_@PDA NPs was weaker, but the tumor was not suppressed. This is because the injection of Mn_3_O_4_@PDA NPs alone can only have a tumor treatment effect through CDT. The tumors of mice injected with MPG NPs became significantly smaller because the surface of GMBP1 gave MPG NPs better tumor targeting ability, causing more MPG NPs to accumulate in the tumor site to achieve a better CDT effect. After the injection of MPG NPs and laser irradiation, the tumor bioluminescence signal of mice was significantly reduced, the tumor gradually decreased, and the tumor disappeared finally. This is because the Mn_3_O_4_ NPs exert a good photothermal effect under laser irradiation. Surface-wrapped PDA also enhances the PTT effect and combines with the CDT effect of MPG NPs to achieve a better synergistic therapy effect. The quantitative signal intensity of bioluminescence is shown in Additional file 1: Fig. S4A–E. Figure 8D shows a picture of the tumor after anatomy. The tumors in the MPG NPs + laser group were the smallest, and among the 5 experimental mice, the tumors in 2 mice disappeared and recovered completely. The quantitative results of tumor weight in Additional file 1: Fig. S4F also showed the same results. The tumor weight of the MPG NPs + laser group was significantly different from that of the other groups. During the treatment period, the body weight changes of the mice in each group were monitored. As shown in Additional file 1: Fig. S4G, the mice in the PBS group were weakened due to the gradual enlargement of tumors, which led to significant weight loss. The weight of the mice in the MPG NPs + laser group also gradually increased due to the recovery of their physical condition. These results indicate that MPG NPs have a good synergistic therapeutic effect. The tumors of each group of mice were sectioned and stained for observation. As shown in Fig. 8E, the H&E staining results showed that the tumor cell structure of the mice in the MPG NPs + laser group was destroyed and showed a necrotic form. The results of TUNEL staining (Fig. 8E and Additional file 1: Fig. S4H) showed that the tumor apoptosis of mice in the MPG NPs + laser group was the most severe, which was consistent with the results of in vivo synergistic therapy. All of the above results show that MPG NPs have good synergistic therapy effect and can be used for CDT/PTT synergistic therapy for gastric cancer MDR.

### In vivo biosafety assessment

To investigate the in vivo toxicity of MPG NPs, acute toxicity and hemolysis tests were carried out. Additional file 1: Fig. S5A shows the hemolysis rate of various materials. Among them, the hemolysis rate of Mn_3_O_4_@PEG NPs is approximately 10%, which indicates certain blood toxicity. The hemolysis rates of MPG NPs, PDA and GMBP1 were 1.8%, 2% and 0.7%, respectively. The results show that they are almost nontoxic, further indicating that MPG NPs can be used for in vivo studies. Then, an in vivo acute toxicity test was carried out. Three groups of healthy mice were injected with GMBP1, Mn_3_O_4_@PDA NPs and MPG NPs at a concentration of 50 mg kg^-1^. The survival and body weight changes of the mice within 14 days were observed. The results showed that all 30 mice survived after 14 days. The body weight of each group of mice shown in Additional file 1: Fig. S5B exhibits a steady upward trend. The pathological changes of mice in each group were recorded within 14 days. As shown in Additional file 1: Fig. S5C, the mice in each group showed pathological changes to a certain extent, such as coarse hair, bulgy eyeballs, and bradykinesia, but this situation was improved after a week, this may be due to the toxicity of high concentrations of manganese ions. The specific statistical results of the pathological characteristics of mice in each group are shown in Additional file 1: Table S1. Among them, the pathological characteristics of mice in the Mn_3_O_4_@PDA NP treatment group were more obvious than those in the GMBP1 and MPG NP treatment groups. The pathological section in Additional file 1: Fig. S5D shows that GMBP1, Mn_3_O_4_@PDA NPs and MPG NPs exerted no obvious damage on the heart, liver, or kidneys of mice. This is due to the good tumor cell targeting of MPG NPs, which allows them to accumulate more in the tumor site, avoiding damage to normal tissues. The above results all indicate that MPG NPs have good biosafety.

## Conclusions

Postoperative chemotherapy of gastric cancer easily causes MDR, which seriously affects the therapeutic effect. Therefore, accurate monitoring of MDR is very important for the treatment of gastric cancer. In past studies, existing MDR detection methods could not reflect the real state of MDR well to some extent. Therefore, it is necessary to explore new methods for real-time monitoring of MDR. Once MDR is accurately detected, individualized treatment can be performed on patients to greatly improve the survival rate. In view of this, a GMBP1 cross-linked TME-responsive self-enhanced Mn_3_O_4_ nanoplatform (MPG NPs) was established, which can react with GSH in tumor cells to produce Mn^2+^ to enhance T1-weighted MRI and mediate in vivo MDR monitoring. In vitro MRI results showed MRI signal enhancement activated by acidic pH, H_2_O_2_ and GSH. In vivo MRI results also showed that MPG NPs can specifically target SGC 7901 ADR tumors, which indicated that MPG NPs can be used for in vivo MDR monitoring. Furthermore, MPG NPs have good chemodynamic activity, which can convert the endogenous H_2_O_2_ of tumor cells into highly toxic ·OH through a Fenton-like reaction at acidic pH to play the role of CDT. In addition, Mn_3_O_4_ NPs can be used as photothermal conversion agents for tumor PTT, which significantly enhances the CDT effect. A series of in vivo and in vitro experiments have shown that MPG NPs demonstrate a good inhibitory effect and synergistic therapeutic effect on tumor cells. Furthermore, MPG NPs have good biocompatibility, providing a good nanoplatform for real-time monitoring and precise diagnosis and treatment of gastric cancer MDR.

## Materials and methods

### Reagents

Manganese acetate (Mn(CH_3_COO)_2_, 98%), DSPE-PEG_2000_-NH_2_ and reduced L-glutathione (GSH) were purchased from Sigma–Aldrich. Oleic acid, oleylamine, dopamine hydrochloride, methylene blue (MB), 1-(3-dimethylaminopropyl)-3-ethylcarbodiimide hydrochloride (EDC), fluorescein diacetate (FDA) and paraformaldehyde (4%) were purchased from Aladdin. 4′,6-Diamidino-2-phenylindole (DAPI) was purchased from Boster Biological Technology. 2,7-Dichlorodihydrofluorescein diacetate (DCFH-DA) was purchased from Meilunbio. D-luciferin potassium salt was purchased from Sciencelight. Propidium iodide (PI) was purchased from Beyotime Biotechnology. The H&E staining kit and Annexin V-FITC apoptosis detection kit were purchased from Solarbio. A terminal-deoxynucleotidyl transferase-mediated nick end labeling (TUNEL) staining kit was purchased from Servicebio. Vinyl-modified GMBP1 (GMBP1-Ack) was synthesized by GL Biochem (Shanghai) Ltd.

### Preparation of MPG NPs

Mn_3_O_4_ NPs were synthesized according to a previous thermal decomposition method [30]. Manganese acetate (0.17 g), oleic acid (640 µL) and oleylamine (3.28 mL) were mixed and dissolved in xylene (15 mL). The reaction was stirred at 90 °C for 10 min, and then 1 mL of DI water was added to react for an additional 2.5 h. After the reaction, 40 mL of absolute ethanol was added, and the precipitate was collected by centrifugation and dispersed in cyclohexane to obtain Mn_3_O_4_ NPs. Mn_3_O_4_@PDA NPs were synthesized according to a previous method [45]: 300 µL of Tween 20 was added dropwise to 20 mL of DI water and stirred at room temperature for 30 min. Next, 500 µL of Mn_3_O_4_ dispersed in cyclohexane (15.6 mg mL^− 1^) was added and sonicated for 1 h until the solution was clear. 2 mL of dopamine hydrochloride solution (1 mg mL^− 1^, pH 8.5 tris HCl) was added and stirred overnight at room temperature. The precipitate (Mn_3_O_4_@PDA NPs) was collected by centrifugation (14,000 rpm, 15 °C, 2 h) and dispersed in 1.56 mL of DI water.

The tetrazole compound N-(2-aminoethyl)-4-(2-(4-methoxyphenyl)-2 H-tetrazol-5-yl)-benzamide (T) was synthesized according to a previous method [46]: 5 mmol methyl 4-formylbenzoate dissolved in 60 mL of absolute ethanol. An equivalent amount of benzenesulfonyl hydrazide was added and reacted overnight. The product was filtered with suction and washed with absolute ethanol. The product was dried to obtain Schiff base. 4-Methoxybenzeneamine at 2.5 mmol was dissolved in 5 mL of a mixed solution of absolute ethanol and water (1:1). Then, 0.5 mL of concentrated hydrochloric acid was slowly added dropwise under an ice bath and stirred for 10 min. An aqueous solution of 2.6 mmol sodium nitrite was slowly added dropwise, and stirring was continued for 1 h under an ice bath. At the end of the reaction, the solution was slowly added to a pyridine solution containing 2.5 mmol Schiff base and reacted overnight. After adding an equal volume of water, a large amount of solids precipitated out, were filtered and washed with a mixed solution of ether and ethyl acetate to obtain a pink solid, which was dried for further use. Then, 0.1 mmol of pink solid was suspended in 10 mL of ethylenediamine and reacted in a water bath at 80 °C for 12 h. The product was concentrated under reduced pressure and purified by a column with methanol and dichloromethane to obtain the product T. The DMSO solution (2.13 µg mL^-1^, 200 µL) of the tetrazole compound T was added dropwise to the Mn_3_O_4_@PDA NP aqueous solution (5 mg mL^-1^, 200 µL) and reacted overnight at room temperature. The reaction solution was purified by PD-10 to obtain Mn_3_O_4_@PDA-T NPs. The peptide GMBP1 was added to the Mn_3_O_4_@PDA-T NP solution (T:GMBP1 = 1:10) and irradiated with a 254 nm portable UV lamp for 2 h. The mixture was purified by PD-10 to obtain MPG NPs.

### Preparation of Mn_3_O_4_@PEG NPs

Mn_3_O_4_@PEG NPs were synthesized according to the literature [30]. One milliliter of Mn_3_O_4_ NPs (cyclohexane) was added to 1 mL of absolute ethanol, and the precipitate was left after centrifugation and dispersed in 3 mL of chloroform. Then, 25 mg of DSPE-PEG_2000_-NH_2_ was added and reacted at room temperature for 2 h. The chloroform was dried with N_2_, and the product was quickly dissolved in 10 mL of DI water to obtain Mn_3_O_4_@PEG NPs.

### Characterization of MPG NPs

The successful synthesis of Mn_3_O_4_ NPs was characterized by XRD and TEM images. The synthesis of Mn_3_O_4_@PDA NPs was characterized by TEM images. The hydration particle size and zeta potential data were obtained by a Malvern particle size analyzer. The synthesis of tetrazolium compound T was characterized by mass spectrometry, nuclear magnetism, ultraviolet absorption spectroscopy and fluorescence spectroscopy. The successful synthesis of MPG NPs was characterized by ultraviolet absorption spectroscopy and fluorescence spectroscopy. The MRI images and T1 relaxation rates of Mn_3_O_4_ NPs and Mn_3_O_4_@PDA NPs under different conditions were obtained by a magnetic resonance imager (Numai). The in vitro stability of MPG NPs was measured by the change in particle size of samples dispersed in PBS and 10% FBS within 6 days at different temperatures (25 and 37 °C).

### In vitro fluorescence imaging of MPG NPs

A PerkinElmer IVIS system was used to detect the fluorescence signal of MPG NPs. The laser excitation wavelength was 420 nm, and the emission wavelength was 570 nm. MPG NPs were diluted into different concentration gradients and placed in a black 96-well plate to obtain fluorescence images corresponding to different concentrations. The fluorescence intensity values of different concentrations were extracted for normalization.

### T1-weighted MRI of MPG NPs and determination of relaxation constant r1

The T1 relaxation time and T1-weighted images of MPG NPs and Mn_3_O_4_@PDA NPs were measured with a 0.5 T small animal magnetic resonance scanner. The measurement method was as follows: MPG NPs or Mn_3_O_4_@PDA NPs were diluted into 5 concentration gradients and placed in a 200 µL centrifuge tube to obtain the T1 relaxation time corresponding to different concentrations. The parameters of the conventional spin echo acquisition sequence were set as follows: TR = 400 ms, TE = 18.2 ms, slice gap = 0.8 mm, slice width = 3 mm. The value of r1 was calculated by linear fitting of 1/T1 (s^− 1^) to the concentration of Mn_3_O_4_ NPs (mM).

To verify the T1-weighted MRI properties of MPG NPs, relaxation images and relaxation time were acquired using an MRI instrument. The relaxation images and relaxation time of MPG NPs at different concentrations and different pH values (pH 7.4, pH 6, pH 5) were determined, and a line graph of the relaxation rate according to the relaxation time was drawn. DI water samples without MPG NPs served as controls. To verify that the increase in Mn^2+^ can enhance T1-weighted MRI, the relaxation images and relaxation time of MPG NP samples with or without H_2_O_2_ were measured. MPG NP solutions of different Mn concentrations were mixed with GSH and reacted for 10 min to obtain MRI images and relaxation times.

### In vitro and in vivo photothermal effect of MPG NPs

The photothermal effect of MPG NPs was verified by measuring the changes in temperature of different materials, different irradiation powers, and different concentrations. First, the temperature changes of different samples (H_2_O, Mn_3_O_4_@PEG NPs, PDA, MPG NPs) with the same concentration were measured after 5 min of 808 nm laser irradiation. Then, the temperature changes of the same concentration of Mn_3_O_4_@PEG NPs and MPG NPs under different powers (0.5 W, 1 W, 2 W) of the 808 nm laser were measured. Finally, the temperature changes of different concentrations of Mn_3_O_4_@PEG NPs and MPG NPs were measured after 808 nm laser irradiation for 5 min. In addition, an 808 nm laser was used to irradiate MPG NPs for 5 min, the temperature was then lowered to room temperature, and 5 cycles were carried out to observe the photothermal stability of MPG NPs.

An orthotopic mouse model of SGC 7901 ADR was established. The mice in the control group were injected with 200 µL of PBS intravenously, and the mice in the experimental group were injected with 200 µL of Mn_3_O_4_@PDA NPs and 200 µL of MPG NPs (the concentration of Mn_3_O_4_ was 5 mg mL^-1^). After 6 h, the tumor site was irradiated with an 808 nm laser (2 W) for 3 min, and photothermal images of mice in each group were obtained.

### ·OH generation by Mn^2+^-mediated Fenton-like reaction

A 25 mM NaHCO_3_/5% CO_2_ buffer solution containing methylene blue (MB, 10 µg mL^-1^), H_2_O_2_ (8 mM), and Mn(CH_3_COO)_2_ (0.5 mM) was reacted at 37 °C for 30 min. The UV absorption spectrum of the sample was measured with a UV spectrophotometer to verify that the ·OH generated by the Fenton-like reaction induced MB degradation. In addition, different concentrations of H_2_O_2_ and Mn^2+^ were used to obtain the UV absorption spectrum of MB.

### The scavenging effect of GSH on ·OH

In the mixture of MB (10 µg mL^-1^), H_2_O_2_ (8 mM), Mn(CH_3_COO)_2_ (0.5 mM) and 25 mM NaHCO_3_/5% CO_2_, GSH (10 mM) was added or not to observe the degradation of MB and verify the scavenging effect of GSH on ·OH. In addition, different concentrations of GSH (0–10 mM) were used to verify the scavenging effect of GSH on ·OH.

### Chemodynamic activity of Mn_3_O_4_@PEG NPs and MPG NPs

Different concentrations of GSH were added to the Mn_3_O_4_@PEG NP solution, the color change of the solution was observed, and the ability of GSH to react quickly with Mn_3_O_4_ to generate Mn^2+^ was verified. In the mixture of MB (10 µg mL^-1^), H_2_O_2_ (8 mM), Mn_3_O_4_@PEG NPs ([Mn] = 0.5 mM), MPG NPs ([Mn] = 0.5 mM) and 25 mM NaHCO_3_/5% CO_2_, different concentrations of GSH were added to observe the degradation of MB.

### In vitro ROS production

SGC 7901 ADR cells were placed in a culture flask for culture, the medium was changed to 50-60%, and the corresponding materials were added to continue culturing for 12 h in a 37 °C incubator. The groups were divided as follows: (1) control; (2) Mn(CH_3_COO)_2_: 20 µg mL^-1^; (3) MPG NPs: 10 µg mL^-1^; and (4) MPG NPs: 20 µg mL^-1^. After 12 h, the medium was removed, and the cells were trypsinized and washed with 1 mL of PBS. DCFH-DA solution (10 µM) was added to each group and incubated for 30 min at 37 °C. The supernatant was removed by centrifugation, and the cells were dispersed in 300 µL of PBS. The suspended cells were dropped into a confocal dish, and the fluorescence of DCF was observed by confocal microscopy. The MFI was quantified by Image J software.

### Cell lines and orthotopic gastric cancer mice model

Luciferase-labeled gastric cancer cell lines (SGC 7901-Luc and SGC 7901 ADR-Luc) were donated by Xijing Hospital. All cells were cultured in 10% FBS medium and incubated at 37 °C in a 5% CO_2_ atmosphere. Severe combined immunodeficiency (SCID) mice (4 weeks old, approximately 16 g) were purchased from the Experimental Center of Xi’an Jiaotong University. Mice were fed under SPF conditions. An orthotopic mouse model of gastric cancer was used for in vivo MRI and synergistic therapy. 100 µL of SGC 7901-Luc or SGC 7901 ADR-Luc cells (5 × 10^6^ cells/mL) was injected into the stomach wall of mice through surgery. After injection, mice were fed under SPF conditions. The size of the tumor was monitored by bioluminescence imaging. All animals were kept in accordance with the Guidelines for Use and Care of Animals at Xi’an Jiaotong University (Number XJTULAC 2016 − 412).

### Cellular uptake and affinity analysis

SGC 7901 ADR cells in the logarithmic growth phase were seeded in a confocal dish (1 × 10^5^ cell/well) and cultured for 48 h. The medium was removed, and 1 mL of PBS solution containing MPG NPs was added after the cells adhered to the wall. The cells were incubated at 37 °C for different times (1 h, 3 h, 6 h, 9 h). The medium was removed and washed with PBS. Then, 1 mL of 4% paraformaldehyde was quickly added to fix the cells for 20 min. The solution was removed, and the cells were washed 3 times with water. Then, 500 µL of DAPI was added for staining for 15 min. The cells were washed with water 3 times to remove the DAPI dye solution and observed qualitatively with a confocal microscope. For the affinity experiment, the adherent cells were divided into 4 groups. The first group was SGC 7901 ADR cells, which were incubated with MPG NPs for 6 h. The second group was SGC 7901 cells, which were incubated with MPG NPs for 6 h. The third and fourth groups were SGC 7901 cells and SGC 7901 ADR cells, respectively, which were incubated with GMBP1 for 6 h first and then incubated with MPG NPs for an additional 6 h. The nucleus was stained with DAPI and qualitatively observed with a confocal microscope. All confocal images were quantitatively analyzed using Image J software.

### Cell inhibition of MPG NPs

The cell suspension (3 cell lines) was added to 96-well plates and incubated for 24 h (37 °C, 5% CO_2_), 100 µL per well. Samples (Mn_3_O_4_@PDA NPs, T, GMBP1, Mn_3_O_4_@PDA-T NPs, MPG NPs) of different concentrations were added to different wells and incubated for 24 h. The medium was removed, the cells were washed 3 times with PBS, and new medium was added. Next, 10 µL of MTT solution was added to each well. After incubation for 2 h, a microplate reader was used to measure the absorbance at 490 nm. The cell survival rate was calculated according to the formula:

1 $$\text{Cell viability}=\frac{{\text{OD}}_{\text{MPG NPs}}-{\text{OD}}_{\text{black}}}{{\text{OD}}_{\text{control}}-{\text{OD}}_{\text{black}}} \times 100\%$$

To verify the effect of laser irradiation on the cell survival rate, the cells were incubated with different concentrations of MPG NPs for 3 h and then irradiated with an 808 nm laser for 5 min (2 W, 5 cm distance). Cells treated without MPG NPs were used as controls. The cells were incubated for 24 h. The cell survival rate was calculated.

### Colony formation

The inhibitory effect of MPG NPs was evaluated by colony formation experiments. The cells were seeded in a 6 cm petri dish containing 10 mL of prewarmed medium, with 500 cells per well. Different concentrations of MPG NPs were added to different petri dishes, and petri dishes with DMSO were added as controls. The cells were irradiated with an 808 nm laser for 5 min (irradiation distance 5 cm). The petri dish was placed in an incubator at 37 °C and incubated for 48 h. The medium was changed, and the cells were incubated for 5 days (37 °C, 5% CO_2_). The medium was removed, and the cells were carefully washed twice with PBS. Then, 4% paraformaldehyde solution was added to fix the cells for 15 min. The solution was removed, and crystal violet staining solution was quickly added. After dyeing, the excess dye solution was rinsed slowly with DI water. After the petri dish was dry, the cells were photographed and counted.

### Flow cytometry

Flow cytometry was used to detect cell apoptosis after treatment with different concentrations of MPG NPs. The cells were seeded in 24-well plates, and different concentrations of MPG NPs were added. An 808 nm laser was used to irradiate the cells for 2 min with a power of 2 W. The cells treated without MPG NPs were used as a control. The cells were placed in an incubator and incubated for 6–8 h. The cells were collected and washed with PBS and then resuspended in PBS. The cells were stained with Annexin-V FITC for 1 h and then stained with PI for 30 min. A FACScan system was used to analyze the stained cells.

### In vitro photothermal effect of MPG NPs

SGC 7901 ADR cells were inoculated in 96-well plates, the medium was removed when the cells grew to 50-60%, and the cells were washed once with PBS. MPG NP solution was added (final concentration of 100 µM). Cells incubated without MPG NPs were used as controls. The cells in the experimental group were irradiated with an 808 nm laser for 3 min and then cultured in an incubator for 8 h. The culture medium was removed, and the cells were washed once with PBS and stained with FDA and PI. FDA (5 µg mL^-1^) and PI (5 µg mL^-1^) solution were added to each well and incubated for 10 min at 37 °C in the dark. After incubation, fluorescent staining was observed under a fluorescence microscope. FDA-positive cells showed green fluorescence, and PI-positive cells showed red fluorescence.

### In vivo T1-weighted MRI and immunofluorescence staining

SGC 7901 ADR-Luc and SGC 7901-Luc orthotopic tumor-bearing mouse models were established. The tumor size was monitored by bioluminescence imaging. Mice were intraperitoneally injected with luciferase substrate (75 mg kg^-1^), and bioluminescence imaging was performed 10 min later to observe the tumor size. In vivo T1-weighted MRI was performed when the tumor grew to an appropriate size. The tumor-bearing mice were divided into four groups (n = 3). The mice were placed in a magnetic resonance imager (Philips ingenia 3.0 T) for imaging, and MRI images were obtained. Mice in different experimental groups were injected with different nanomaterials. SGC 7901 ADR tumor-bearing mice and SGC 7901 tumor-bearing mice were injected intravenously with MPG NPs (50 mg kg^-1^). Mice in the blocking group were injected with 100 µL of GMBP1 (1 mg mL^-1^) through the tail vein first and then injected with MPG NPs (50 mg kg^-1^) half an hour later. In vivo MRI was performed at different times (1 h, 2 h, 3 h, 4 h, 5 h) after injection, and MRI images before the injection were also obtained. Signal extraction was performed on the tumor area, and the signal changes at the tumor site were quantitatively observed. After in vivo imaging, the main organs (heart, liver, and kidney) and tumor tissues of each group of mice were collected, frozen and sectioned, and histological analysis was performed by conventional immunofluorescence staining [47]. Rabbit anti-human GRP78 and FITC-labeled goat anti-rabbit antibodies were used for MPG NP visualization, and the nuclei were labeled with DAPI. CLSM was used to observe tissue sections.

### In vivo CDT/PTT synergistic therapy

Twenty-five orthotopic tumor-bearing mice were randomly divided into 5 groups (n = 5) as follows: (1) PBS; (2) PBS + laser; (3) Mn_3_O_4_@PDA NPs; (4) MPG NPs; and (5) MPG NPs + laser. Each mouse was monitored for tumor growth by bioluminescence imaging. When the tumor grew to a suitable size, in vivo synergistic therapy was conducted. Mice in different groups were injected with the corresponding nanomaterials (50 mg kg^-1^) and received in vivo synergistic therapy, with intervention every three days. Six hours after the injection of the corresponding nanomaterials, the mice in the PBS + laser group and MPG NPs + laser group were irradiated with an 808 nm laser (2 W) to the tumor sites for 3 min for PTT (irradiation distance was 5 cm). After 12 h, all groups of mice were subjected to bioluminescence imaging, and the body weight data of each mouse were obtained. The changes in tumors were continuously monitored for 20 days. After 20 days, the mice in each group were sacrificed, and tumor tissues of the mice were obtained, photographed, fixed with 4% paraformaldehyde, and then paraffin sectioned. H&E staining was performed on the tumors of mice, and TUNEL staining was performed to observe the apoptosis of tumor cells.

### In vivo acute toxicity and histological staining

Thirty healthy mice (15 female mice and 15 male mice) were randomly divided into three groups, and each group contained 5 female mice and 5 male mice. The first group of mice was injected with 200 µL of GMBP1 (1 mg mL^-1^) as a control, the second group of mice was injected with 200 µL of Mn_3_O_4_@PDA NPs (50 mg kg^-1^), and the third group of mice was injected with 200 µL of MPG NPs (50 mg kg^-1^). The body weight and death of each group of mice within 14 days were recorded, and the survival rate of the mice was calculated. After 14 days, the mice were dissected, and the major organs of the mice were taken for H&E staining.

### Hemolysis test

Whole blood of mice was obtained and added to Triton X-100 (1% V/V), Mn_3_O_4_@PEG NPs, MPG NPs, PDA, and GMBP1 and incubated at 37 °C for 2 h. The blood samples were centrifuged at 13,000 rpm for 15 min, the supernatant was taken, the absorbance at 540 nm was measured with an ultraviolet spectrophotometer, and the hemolysis rate was calculated.

### Statistical analysis

The data represent three or more independent experiments. Statistical analysis was performed using analysis of variance. p < 0.05 was considered indicative of a significant difference. All statistical analyses were performed using GraphPad Prism software.

## Supplementary Information

**Additional file 1: FigureS1. **Synthesis and characterization of tetrazole compound. (A) Synthesis of tetrazole compound. (B) Mass spectrum of tetrazolium compound. (C) ^13^C NMR of tetrazolium compound. **Figure S2. **In vitro chemodynamic activity of MPG NPs. (A) The remaining percent of MB after different treatments. (B) Photographs of Mn_3_O_4_@PEG NPs after exposure to different concentrations of GSH. (C) Photographs of Mn_3_O_4_@PEG NPs reacted with GSH for 5 min and dispersed in aqueous solutions of different pH for different times. (D) The remaining percent of MB after treatment with different concentrations of MPG NPs. (E) The remaining percent of MB after different concentrations of Mn_3_O_4_@PEG treatment. (F) MB degradation efficiency after treatment of different samples. (G) Quantification of DCF fluorescence in SGC 7901 ADR cells after different sample treatments. **Figure S3. **Cell uptake and affinity analysis, and in vivo and in vitro quantitative results. (A) Fluorescence images of SGC 7901 ADR cells incubated with MPG NPs for different times. (B) Fluorescence of SGC 7901 and SGC 7901 ADR cells with or without GMBP1 blockade. (C) Quantification of the fluorescence intensity of SGC 7901 ADR cells incubated with MPG NPs for different times. (D) Quantification of the fluorescence intensity of SGC 7901 and SGC 7901 ADR cells incubated with or without GMBP1 blockade. (E) Quantification of in vivo MRI signal intensity. **Figure S4. ** Quantitative results of in vivo synergistic therapy. (A)-(E) Quantification of bioluminescence imaging signal intensity in each group of mice within 20 days. (F) Tumor weight of mice in each group. (G) Changes in body weight of mice in each group within 20 days. (H) Number of TUNEL-positive cells in tumor sections of mice in each group. **Figure S5. ** In vivo toxicity of MPG NPs. (A) Hemolysis rate of Mn_3_O_4_@PEG, PDA, GMBP1 and MPG NPs. (B) Body weight changes of mice within 14 days. (C) Pathological features of mice within 14 days. (D) H&E staining images of the heart, liver and kidney of different groups of mice. **Table S1. ** Statistical analysis of pathological manifestations of mice within 14 days.

## Data Availability

All data generated or analyzed during this study are available in the manuscript or supplementary information.

## Abbreviations

MDR: Multidrug resistance
MRI: Magnetic resonance imaging
CDT: Chemodynamic therapy
TME: Tumor microenvironment
PTT: Photothermal therapy
H_2_O_2_: hydrogen peroxide
·OH: Hydroxyl radicals
NPs: Nanoparticles
MB: Methylene blue
FDA: Fluorescein diacetate
PI: Propidium iodide
MFI: Mean fluorescence intensity
