# Supplementary material for: Tumor microenvironment responsive Mn3O4 nanoplatform for in vivo real-time monitoring of drug resistance and photothermal/chemodynamic synergistic therapy of gastric cancer
Source: J Nanobiotechnology. 2022 May 23;20:240. doi: 10.1186/s12951-022-01441-6 (PMC9125909; doi:10.1186/s12951-022-01441-6)
Supplement: Supplementary file 1 — Additional file 1: FigureS1. Synthesis and characterization of tetrazole compound. (A) Synthesis of tetrazole compound. (B) Mass spectrum of tetrazolium compound. (C) 13C NMR of tetrazolium compound. Figure S2. In vitro chemodynamic activity of MPG NPs. (A) The remaining percent of MB after different treatments. (B) Photographs of Mn3O4@PEG NPs after exposure to different concentrations of GSH. (C) Photographs of Mn3O4@PEG NPs reacted with GSH for 5 min and dispersed in aqueous solutions of different pH for different times. (D) The remaining percent of MB after treatment with different concentrations of MPG NPs. (E) The remaining percent of MB after different concentrations of Mn3O4@PEG treatment. (F) MB degradation efficiency after treatment of different samples. (G) Quantification of DCF fluorescence in SGC 7901 ADR cells after different sample treatments. Figure S3. Cell uptake and affinity analysis, and in vivo and in vitro quantitative results. (A) Fluorescence images of SGC 7901 ADR cells incubated with MPG NPs for different times. (B) Fluorescence of SGC 7901 and SGC 7901 ADR cells with or without GMBP1 blockade. (C) Quantification of the fluorescence intensity of SGC 7901 ADR cells incubated with MPG NPs for different times. (D) Quantification of the fluorescence intensity of SGC 7901 and SGC 7901 ADR cells incubated with or without GMBP1 blockade. (E) Quantification of in vivo MRI signal intensity. Figure S4. Quantitative results of in vivo synergistic therapy. (A)-(E) Quantification of bioluminescence imaging signal intensity in each group of mice within 20 days. (F) Tumor weight of mice in each group. (G) Changes in body weight of mice in each group within 20 days. (H) Number of TUNEL-positive cells in tumor sections of mice in each group. Figure S5. In vivo toxicity of MPG NPs. (A) Hemolysis rate of Mn3O4@PEG, PDA, GMBP1 and MPG NPs. (B) Body weight changes of mice within 14 days. (C) Pathological features of mice within 14 days. (D) H&E staining images [file 12951_2022_1441_MOESM1_ESM.pdf]

Supporting Information

Tumor microenvironment responsive Mn_3O_4 nanoplatform for in vivo real-time monitoring of drug resistance and photothermal/chemodynamic synergistic therapy of gastric cancer

Hanrui Li[1], Xiaoxia Cai[1], Tong Yi[1], Yun Zeng[1], Jingwen Ma[2], Lei Li[2], Liaojun Pang[1], Na Li[2]*, Hao Hu[3]*, Yonghua Zhan[1]*

[1]Engineering Research Center of Molecular & Neuro Imaging of the Ministry of Education, School of Life Science and Technology, Xidian University, Xi’an 710126, China.
[2]Radiology Department, Ninth Affiliated Hospital of Medical College of Xi'an Jiaotong University, Xi’an 710054, China.
[3]Endoscopic Center of Zhongshan Hospital, Fudan University, Shanghai 200032, China.

*Corresponding author.
E-mail: yhzhan@xidian.edu.cn (Yonghua Zhan), hu.hao1@zs-hospital.sh.cn (Hao Hu), ln2002011039@163.com (Na Li).

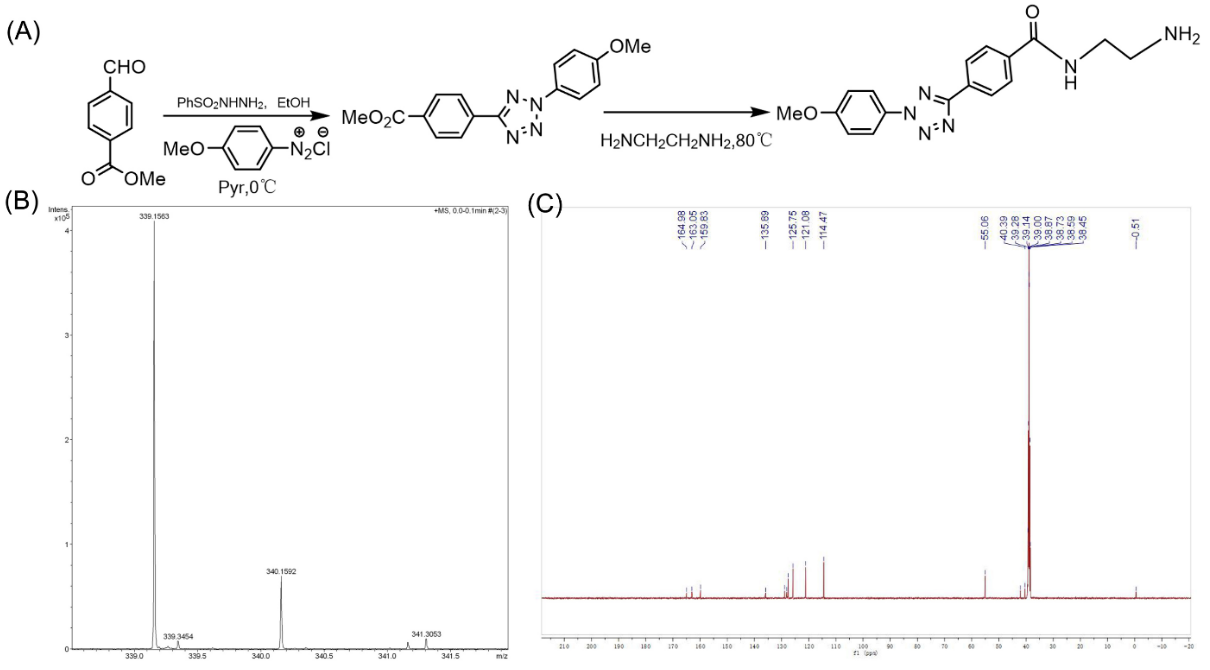

Figure S1. Synthesis and characterization of tetrazole compound. (A) Synthesis of tetrazole compound. (B) Mass spectrum of tetrazolium compound. (C) ^{13}C NMR of tetrazolium compound.

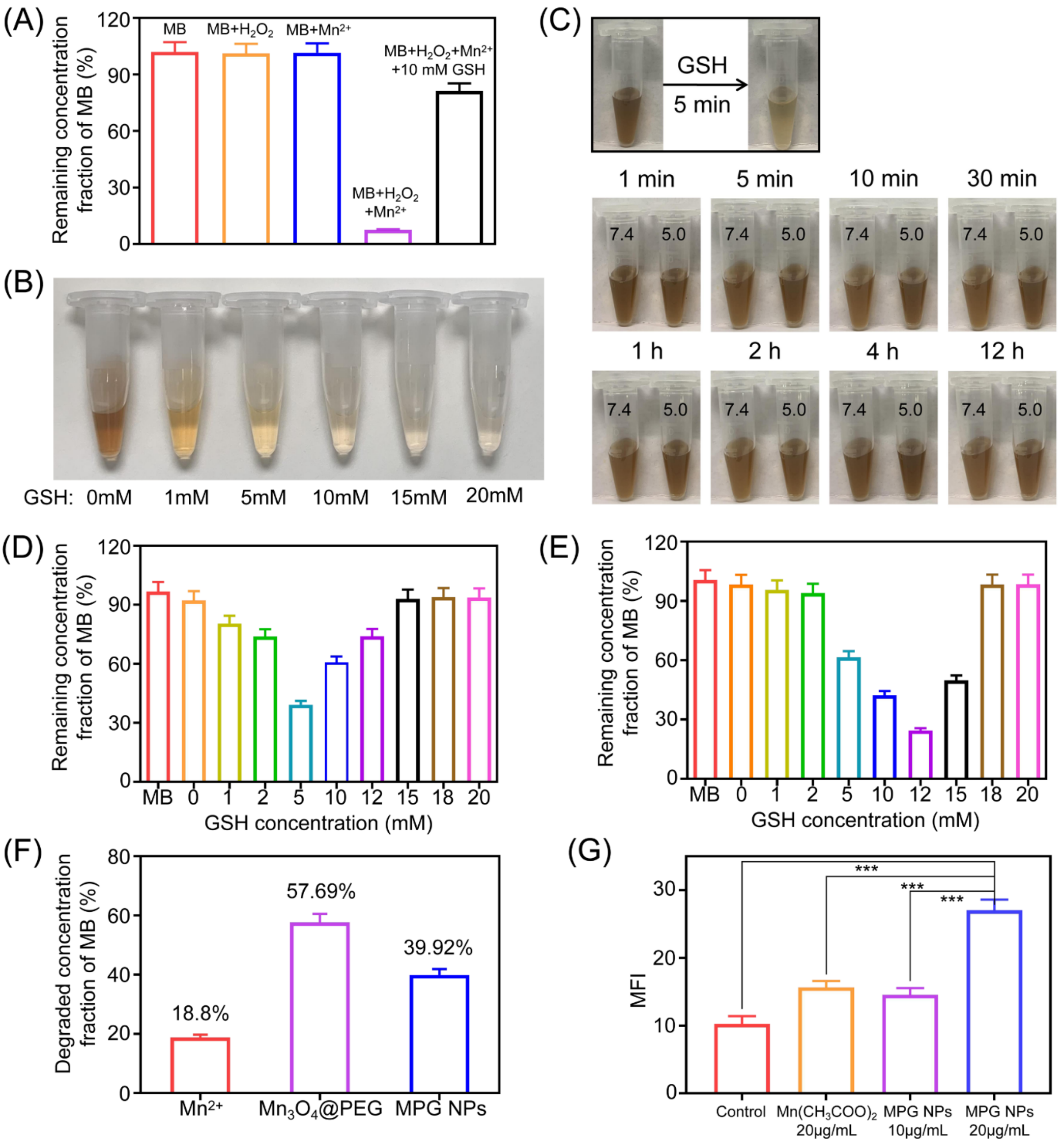

Figure S2. In vitro chemodynamic activity of MPG NPs. (A) The remaining percent of MB after different treatments. (B) Photographs of Mn_3O_4@PEG NPs after exposure to different concentrations of GSH. (C) Photographs of Mn_3O_4@PEG NPs reacted with GSH for 5 min and dispersed in aqueous solutions of different pH for different times (D) The remaining percent of MB after treatment with different concentrations of MPG NPs. (E) The remaining percent of MB after different concentrations of Mn_3O_4@PEG treatment. (F) MB degradation efficiency after treatment of different samples. (G) Quantification of DCF fluorescence in SGC 7901 ADR cells after different sample treatments.

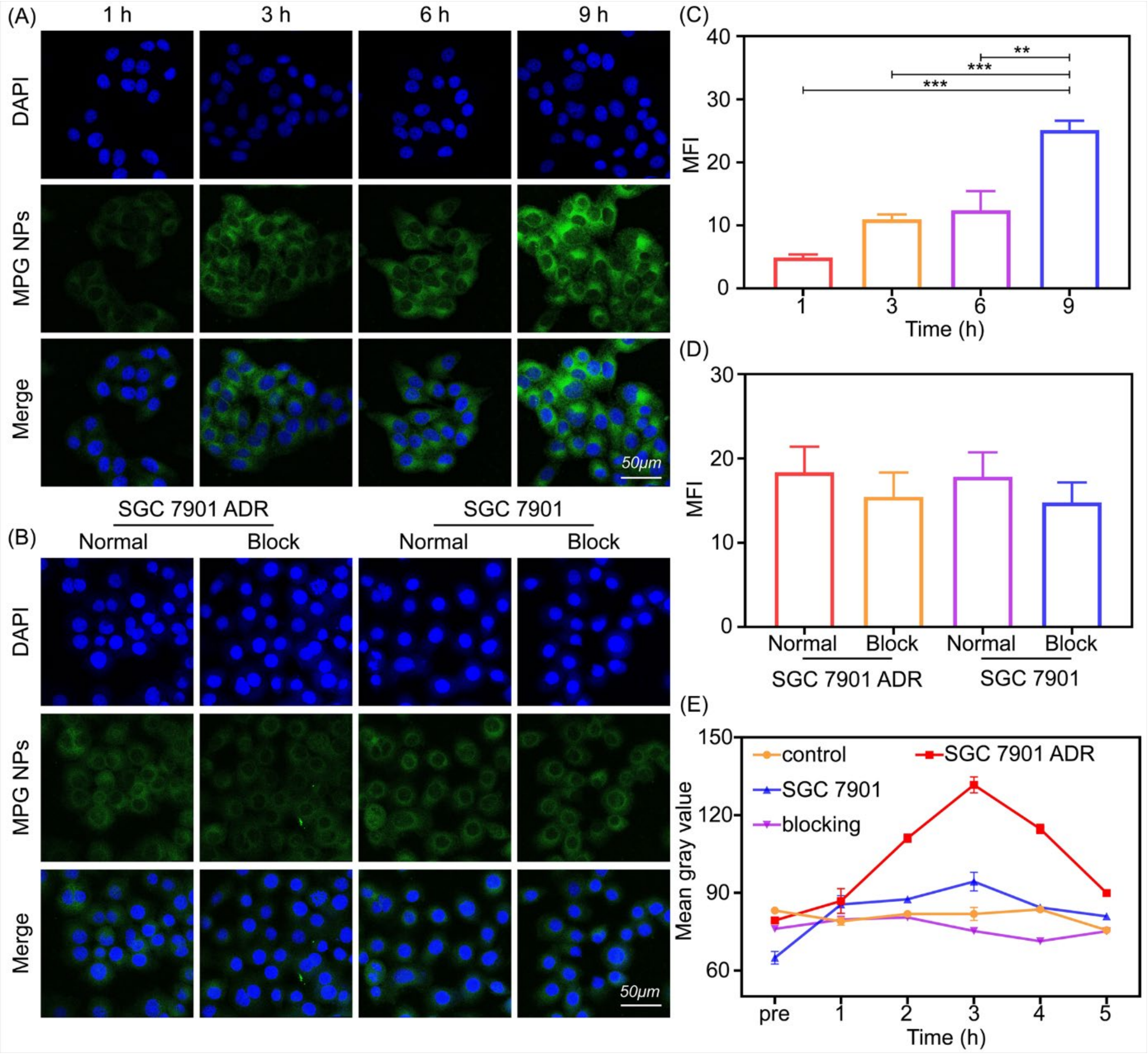

Figure S3. Cell uptake and affinity analysis, and in vivo and in vitro quantitative results. (A) Fluorescence images of SGC 7901 ADR cells incubated with MPG NPs for different times. (B) Fluorescence of SGC 7901 and SGC 7901 ADR cells with or without GMBP1 blockade. (C) Quantification of the fluorescence intensity of SGC 7901 ADR cells incubated with MPG NPs for different times. (D) Quantification of the fluorescence intensity of SGC 7901 and SGC 7901 ADR cells incubated with or without GMBP1 blockade. (E) Quantification of in vivo MRI signal intensity.

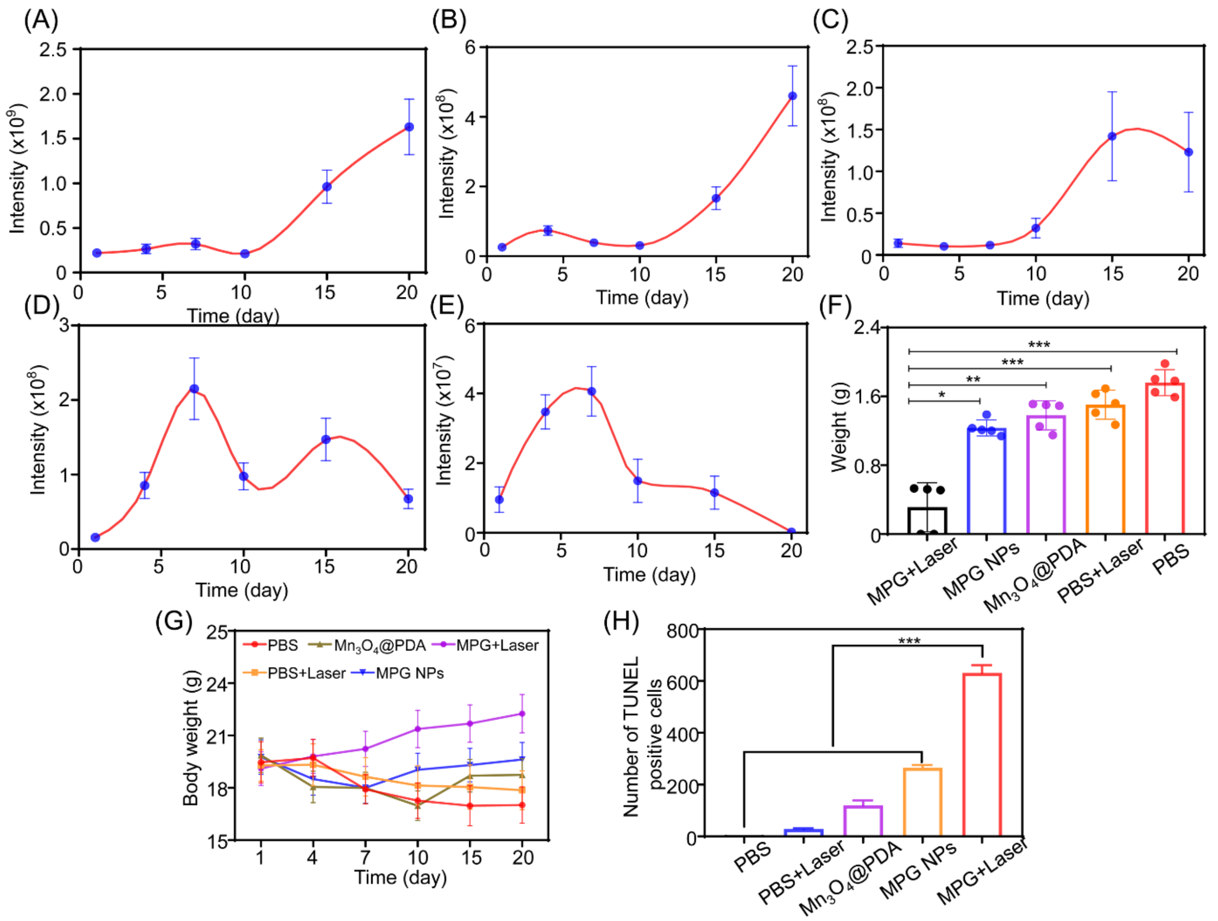

Figure S4. Quantitative results of in vivo synergistic therapy. (A)-(E) Quantification of bioluminescence imaging signal intensity in each group of mice within 20 days. (F) Tumor weight of mice in each group. (G) Changes in body weight of mice in each group within 20 days. (H) Number of TUNEL-positive cells in tumor sections of mice in each group.

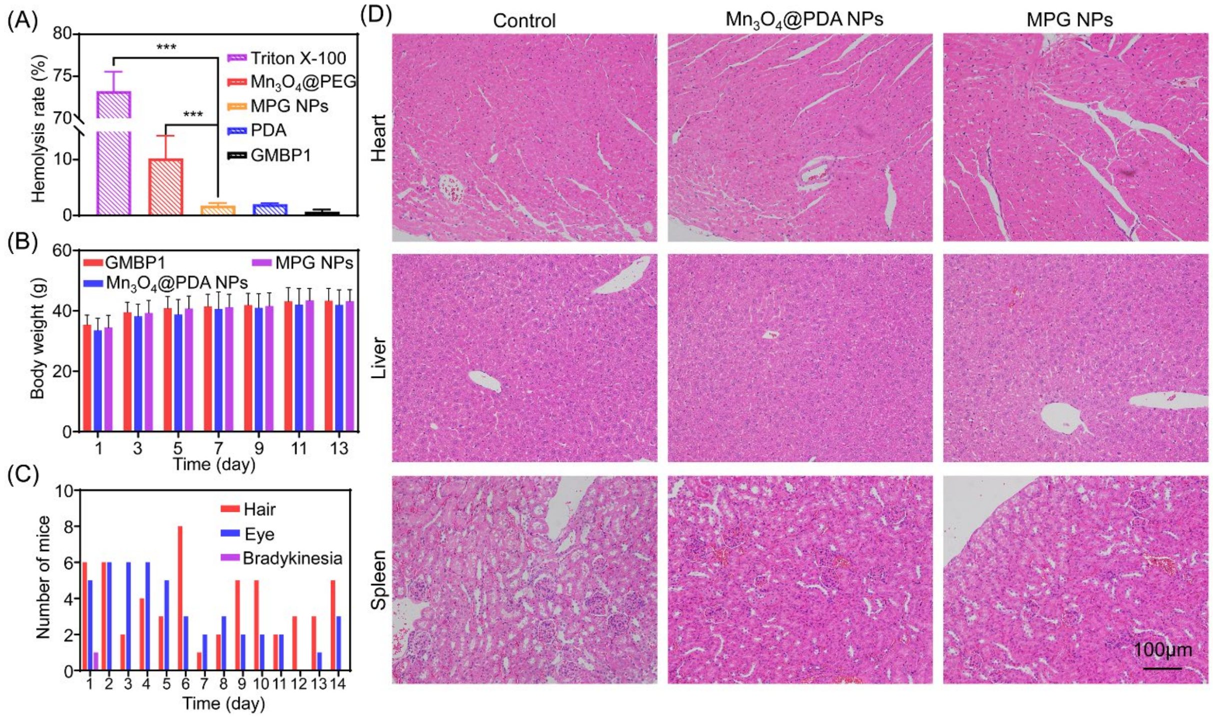

Figure S5. In vivo toxicity of MPG NPs. (A) Hemolysis rate of Mn_3O_4@PEG, PDA, GMBP1 and MPG NPs. (B) Body weight changes of mice within 14 days. (C) Pathological features of mice within 14 days. (D) H&E staining images of the heart, liver and kidney of different groups of mice.

Table S1. Statistical analysis of pathological manifestations of mice within 14 days.

Day		1	2	3	4	5	6	7	8	9	10	11	12	13	14
Characteristics	Groups	The number of mice													
Hair	GMBP1	0	1	0	0	0	0	0	0	0	0	0	0	0	0
	Mn_3O_4@PDA	4	3	2	3	2	4	0	0	4	2	2	2	2	4
	MPG	2	2	0	1	1	4	1	2	1	3	0	1	1	1
Eye	GMBP1	0	1	0	0	0	0	0	0	0	0	0	0	0	0
	Mn_3O_4@PDA	2	3	3	3	1	2	0	2	1	1	1	0	0	2
	MPG	3	2	3	3	4	1	2	1	1	1	1	0	1	1
Bradykinesia	GMBP1	0	0	0	0	0	0	0	0	0	0	0	0	0	0
	Mn_3O_4@PDA	1	0	0	0	0	0	0	0	0	0	0	0	0	0
	MPG	0	0	0	0	0	0	0	0	0	0	0	0	0	0
